# Thrombocytopenia and the effect of platelet transfusions on the occurrence of intracranial hemorrhage in patients with acute leukemia – a nested case-control study

**DOI:** 10.1007/s00277-020-04298-7

**Published:** 2020-10-17

**Authors:** Loes L. Cornelissen, Aukje L. Kreuger, Camila Caram-Deelder, Rutger A. Middelburg, Jean Louis H. Kerkhoffs, Peter A. von dem Borne, Erik A. M. Beckers, Karen M. K. de Vooght, Jürgen Kuball, J. J. Zwaginga, Johanna G. van der Bom

**Affiliations:** 1 Jon J van Rood Center for Clinical Transfusion Medicine, Sanquin/LUMC, Leiden, The Netherlands; 2 Department of Immunology, Leiden University Medical Center, Leiden, The Netherlands; 3 Department of Clinical Epidemiology, Leiden University Medical Center, Leiden, The Netherlands; 4 Department of Hematology, Haga Teaching Hospital, Den Haag, The Netherlands; 5 Department of Hematology, Leiden University Medical Center, Leiden, The Netherlands; 6 Department of Hematology, Maastricht University Medical Center, Maastricht, The Netherlands; 7 Central Diagnostic Laboratory, University Medical Center Utrecht, Utrecht, The Netherlands; 8 Department of Hematology, University Medical Center Utrecht, Utrecht, The Netherlands

**Keywords:** Intracranial hemorrhage, Acute leukemia, Thrombocytopenia, Platelet transfusions, Case-control study

## Abstract

We designed a study to describe the incidence of intracranial hemorrhage according to severity and duration of thrombocytopenia and to quantify the associations of platelet transfusions with intracranial hemorrhage in patients with acute leukemia. In this case-control study nested in a cohort of 859 leukemia patients, cases (*n* = 17) were patients diagnosed with intracranial hemorrhage who were matched with control patients (*n* = 55). We documented platelet counts and transfusions for seven days before the intracranial hemorrhage in cases and in a “matched” week for control patients. Three measures of platelet count exposure were assessed in four potentially important time periods before hemorrhage. Among these leukemia patients, we observed the cumulative incidence of intracranial hemorrhage of 3.5%. Low platelet counts were, especially in the three to seven days preceding intracranial hemorrhage, associated with the incidence of intracranial hemorrhage, although with wide confidence intervals. Platelet transfusions during the week preceding the hemorrhage were associated with higher incidences of intracranial hemorrhage; rate ratios (95% confidence interval) for one or two platelet transfusions and for more than two transfusions compared with none were 4.04 (0.73 to 22.27) and 8.91 (1.53 to 51.73) respectively. Thus, among acute leukemia patients, the risk of intracranial hemorrhage was higher among patients with low platelet counts and after receiving more platelet transfusions. Especially, the latter is likely due to clinical factors leading to increased transfusion needs.

**Electronic supplementary material:**

The online version of this article (10.1007/s00277-020-04298-7) contains supplementary material, which is available to authorized users.

## Introduction

Patients with acute leukemia frequently suffer from bleeding events [1] of which intracranial hemorrhage (ICH) is one of the most serious [2–5]. Reported incidences of (symptomatic) intracranial hemorrhage vary between 2.8% up to 6.1% [2, 5, 6], and fatal intracranial hemorrhages explain more than 50% of fatal bleedings among acute leukemia patients [7].

Acute leukemia patients may develop intracranial hemorrhage due to various causes. Besides risk factors that also play a role in the general population, like age, hypertension, male sex, and ethnicity [8–10], leukemia or cancer-specific risk factors have been established. Among others, these are graft versus host disease, hyperleukocytosis, and thrombocytopenia [11–14]. Of these, the low platelet count is generally considered one of the most important risk factors for bleeding in hemato-oncological patients. It is, however, not conclusively established if, and at what platelet counts, the risk of intracranial hemorrhage increases in this patient population [2, 5–7, 12, 15, 16]. Moreover, prolonged exposure to low platelet counts (≤ 10 × 10^9^/L) may be associated with even higher bleeding risks [17, 18]. We hypothesized that longer periods with low platelet counts as well as lower (through) platelet counts can both determine an increasing risk of intracranial hemorrhage. If these time and trough measures are stronger associated with bleeding risk, this could have implications for future treatment strategies.

To prevent bleeding, hemato-oncology patients with low platelet counts are generally treated with prophylactic platelet transfusions [19–21]. The trigger to transfuse is commonly set at a platelet count of 10 × 10^9^/L [22–24]. Prophylactic platelet transfusions reduced the risk of bleedings in patients with a World Health Organization (WHO) score of ≥ 2 [25] from 50 to 43% [26], with the most benefit for patients with acute myeloid leukemia or intensive chemotherapy treatment [16, 17, 27]. However, the large majority of bleeds is thus not prevented despite platelet transfusions. This raises questions about the causes of bleeding both when patients are treated with prophylactic platelet transfusions and also when they are not. Interestingly, recent high-level evidence suggests that among neonates and among patients with hemorrhagic stroke, both prophylactic and therapeutic platelet transfusions may increase the risk of bleeding and/or mortality and morbidity [28, 29].

How exactly the depth and length of thrombocytopenia and the given platelet transfusions interact and modulate the risk of critical bleeding like intracranial hemorrhage is presently unknown.

Therefore, the objective of this exploratory study was to describe the association of platelet counts assessed in several time periods and severities with the incidence of intracranial hemorrhage in acute leukemia patients. Also, we wanted to examine the association between platelet transfusions and the incidence of intracranial hemorrhage.

## Methods

### Case identification and control selection

We performed a matched case-control study nested in a cohort of patients with acute promyelocytic leukemia, acute myeloid leukemia, acute lymphoblastic leukemia, or myelodysplastic syndrome in four hospitals in the Netherlands. Patients with intracranial hemorrhage were identified via an algorithm based on electronically available health care data [30]. Charts were reviewed to confirm the diagnosis and type of hemorrhage. All patients with confirmed intracranial hemorrhage were potential case patients for our study. Potential cases were excluded if no clinical data was retrievable, the date of bleeding was unclear, it was not the first intracranial hemorrhage, the diagnosis was unclear or unconfirmed, or if there were no eligible control matches possible.

For each case, a minimum of one to a maximum of four control patients was selected from the same cohort, based on availability. The amount of four controls was chosen to ensure optimal power [31]. Controls were matched to case patients according to hospital, diagnosis, and indication for admission. For diagnosis, matching was performed on both the disease, as well as disease status (first diagnosis versus relapsed disease). Control patients with MDS could be matched to a patient with AML if the patient was treated according to an AML protocol, suggesting progression to AML. Matching was performed for several reasons. First, matching allows for correction of risk factors for bleeding that might be difficult to correct for in the unmatched analysis. Second, matching on the hospital was performed to correct for confounders that cannot easily be measured, for example, differences in local treatment protocols.

### Implicated time periods and data collection

**Fig. 1:**
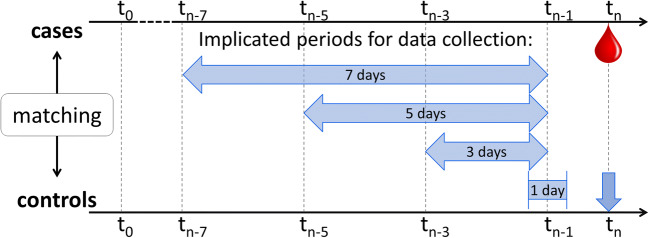
Design: implicated time periods. *t*_0_ = first day of treatment if indication for admission was chemotherapy or stem cell transplantation or day of admission if indication for admission was a complication of former treatment or disease. *t*_n_ = index day: day of intracranial hemorrhage for cases and corresponding day for controls. Matching was performed for hospital, diagnosis and indication for admission

We studied exposures (thrombocytopenia/platelet transfusions) during the week preceding the event of intracranial hemorrhage and defined four potentially implicated time periods within that week: one, three, five and seven days preceding the hemorrhage. Date of bleeding (called “index date”) was defined according to the date of cerebral imaging as well as the date of neurological symptoms or consultation from a neurologist. Figure 1 illustrates the “implicated” periods for control patients, namely the week that coincided with the implicated period of the matched case patient on their timeline since the start of treatment if the patient was currently admitted for chemotherapy or stem cell transplantation. If the admission indication was a complication of former therapy or disease, the implicated period was counted from the first day of the current admission.

We gathered laboratory data, transfusion data, and clinical variables of all cases and controls from the medical files (see online supplementary material).

### Definitions of exposure categories for thrombocytopenia

**Fig. 2:**
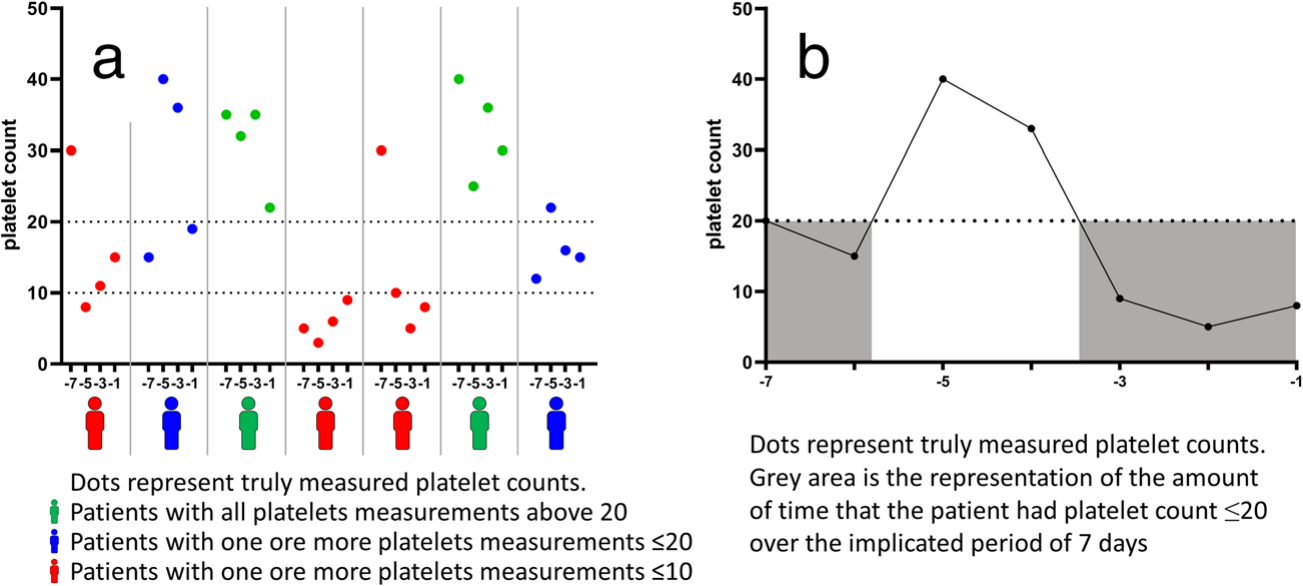
Defined measures of platelet count. Example data, for explanation of the used platelet count measures that are defined. All measures are obtained for all three predefined implicated time periods, but only the 7-day period is graphically presented. Panel a: platelet measure of at least one platelet count ≤ 10 × 10^9^/L or ≤ 20 × 10^9^/L. Dots represent platelet counts at different days. Persons in red have at least one platelet count below the threshold of ≤ 10 × 10^9^/L. Persons in blue have at least one platelet count within ≤ 10 × 10^9^/L and ≤ 20 × 10^9^/L and persons in green have no platelet counts below both thresholds. For the platelet measure of at least one platelet count ≤ 10 × 10^9^/L, persons in red were analyzed as “yes,” and for the platelet measure of at least one platelet count within ≤ 20 × 10^9^/L, persons in red and blue were analyzed as “yes.” Panel b: platelet measure of the percentage of hours with a (expected) platelet count ≤ 20 × 10^9^/L. In the graph, dots represent truly measured platelet counts and the gray areas are the implicated time periods with such a (expected) platelet count. For every patient, a timeline was made of all present platelet counts per implicated time period. We assumed a linear relation between the platelet count within 2 consecutive measurements, lines were therefore interpolated. For every hour between the first and the last measurement of platelet count, the expected measured platelet count was calculated. The percentage of hours with a (expected) platelet count ≤ 20 × 10^9^/L was calculated afterwards. The reason we chose for hours ≤ 20 × 10^9^/L, instead of the more clinical used trigger of hours below ≤ 10 × 10^9^/L, was that we anticipated that the percentage of hours below ≤ 10 × 10^9^/L would be very small since this is a transfusion indication. Thus, it would lead to non-positivity

Three different measures of platelet count were defined to take into account both severity and duration of thrombocytopenia in the potential association between platelet count and intracranial hemorrhage (Fig. 2). These three measures were all assessed for each implicated time period.

- First, the presence of one or more nadir platelet counts of ≤ 10 × 10^9^/L for each implicated period was assessed. As we were studying a seven-day period, a patient with at least one platelet count ≤ 10 × 10^9^/L could have between one and seven low platelet counts; for the five-day period, the value varied between one and five, etc.
- Second, the presence of one or more nadir platelet counts of ≤ 10 × 10^9^/L for each implicated period was investigated.
- Third, we calculated the percentage of hours with a platelet count ≤ 20 × 10^9^/L. All platelet counts measured were put on a timeline. Any change in platelet counts, between two measured platelet counts, was assumed to be linear. Between actual platelet count measurements, for each hour, the expected platelet count was interpolated. This led to an (expected or truly measured) platelet count for each hour a patients was followed. We investigated the percentage of hours with a platelet count below ≤20 x10 /L

We intended to study the percentage of days ≤ 10 × 10^9^/L, but too few patients had several days with platelet count ≤ 10 × 10^9^/L.

### Measures of platelet transfusion

To provide an estimate of the association between platelet transfusions and the occurrence of intracranial hemorrhage, we categorized the number of platelet transfusions (no transfusions, 1–2 transfusions, > 2 transfusions) per period. These categories were selected since for intensively treated patients’ 1–2 platelet transfusions a week were expected to be a normal amount. Also, we explored the sum of platelet transfusions as a continuous variable per implicated period.

### Statistical analyses

We used conditional logistic regression models, which adjusts for matching variables, to assess the associations of the different measures of thrombocytopenia with the incidence of intracranial hemorrhage. In the adjusted analyses, we adjusted for one potential confounding variable at the time. Since incidence-density sampling was used for the selection of controls, odds ratios were interpreted as incidence rate ratios (RR) [32]. Because patients admitted for other indications then chemotherapy or SCT were more likely to have higher platelet counts, a post hoc sensitivity analysis was performed excluding patients who were admitted for another reason then chemotherapy or SCT.

Also, for both categorical and continuous measures of platelet transfusions, conditional logistic regression was performed to assess the association between platelet transfusion and intracranial hemorrhage. This was adjusted for the different measurements of platelet counts that were defined.

Clinical factors can confound the association with intracranial hemorrhage. This was assessed via multivariable conditional logistic regression. The models combined one defined measure of platelet count or platelet transfusion with one clinical variable at a time.

Given that our sample size is small, the analyses are exploratory.

### Ethical considerations

The medical ethical committee of the LUMC waived the need for informed consent (see Compliance with ethical standards).

## Results

### Characteristics of the study population

**Fig. 3:**
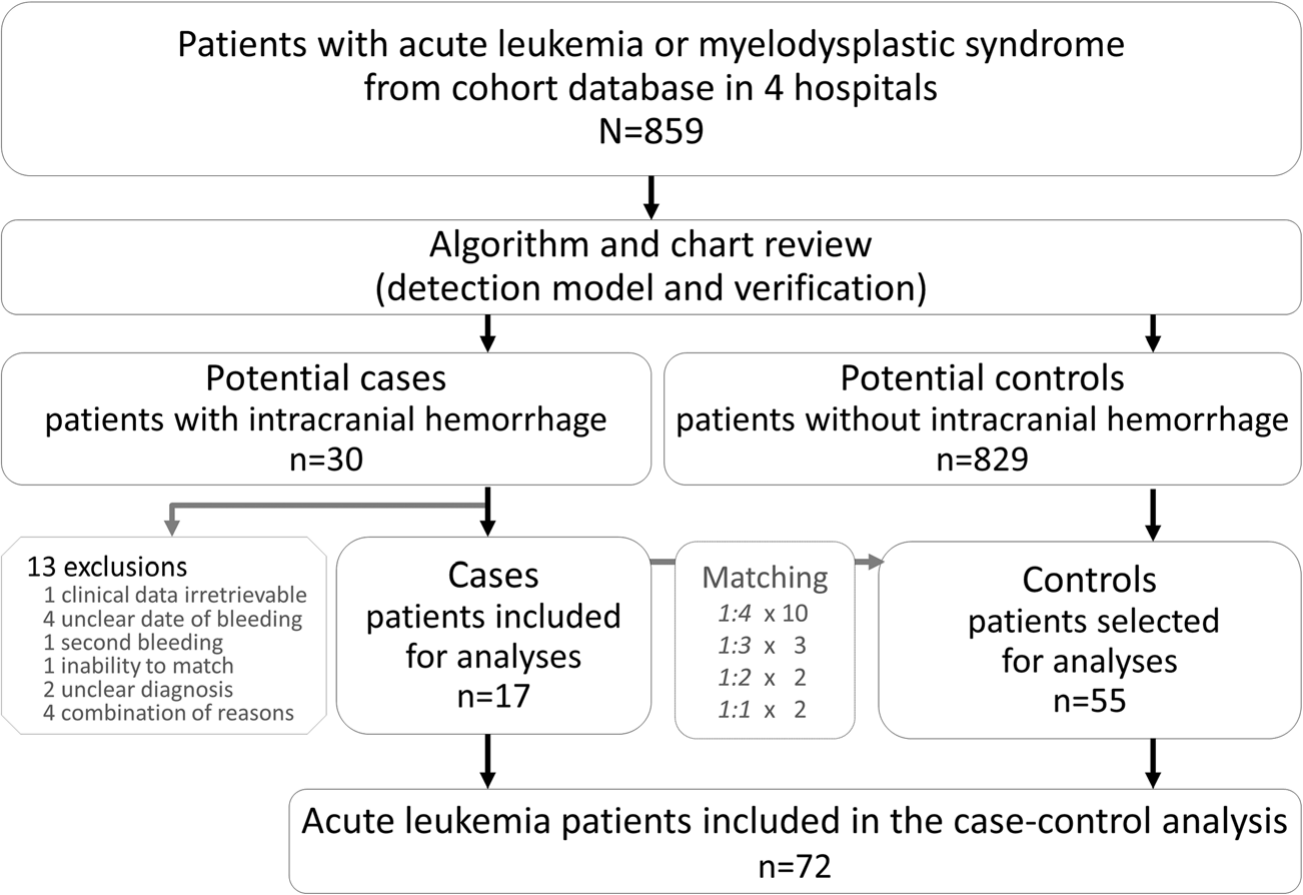
Flowchart. Inclusion period differed per hospital: hospital A June 2011 until March 2017, hospital B January 2010 until December 2015, hospital C January 2010 until December 2015, hospital D Jan 2013 until December 2015. Reasons for exclusion of 13 cases are specified. Unclear date of bleeding was encountered for example when a patient entered the hospital with non-acute neurological symptoms and intracranial hemorrhage was found on the day of admission. One patient excluded for unclear diagnosis never had a pathology result before death; one patient was initially diagnosed as acute leukemia but later classified as lymphoma. One patient had a second intracranial hemorrhage, which already altered transfusion policies. For one case with a double diagnosis of leukemia and intracerebral lymphoma, no eligible match was found. Finally, four patients had a combination of above reasons. If more than four eligible controls were identified, controls closest to the case in calendar time were selected

We identified 30 patients who had suffered an intracranial hemorrhage within the cohort of 859 patients with leukemia (cumulative incidence 3.5%). Thirteen patients had to be excluded from the predefined reasons presented in Fig. 3. Eventually, 72 patients (17 cases and 55 controls) were analyzed in the case-control study.

Distribution of values of matching variables and general characteristics across case and control patients is presented in Table 1. In the case patients, acute myeloid leukemia was the most frequent diagnosis (65%) and with 77%, the most frequent indication for admission was remission induction chemotherapy. The relapsed disease occurred in 29% of case patients; others had the first diagnosis. Due to frequency matching, a direct comparison between these percentages with percentages of matched, selected controls is not appropriate. The type of intracranial hemorrhage was most often intracerebral or subdural (both 35%). One patient suffered a subarachnoid hemorrhage (6%). Of the combined bleedings (24%), three patients had an intracerebral and subarachnoid hemorrhage, and one patient suffered from intracerebral and subdural hemorrhage. Two patients were prescribed tranexamic acid in the seven-day implicated period: one case patient and one control.

**Table 1:** Characteristics of the study population*

Matching variables	Cases *n* = 17	Controls *n* = 55	Total *n* = 72
Diagnosis			
ALL	5 (29)	19 (35)	24 (33)
AML/MDS	11 (65)	35 (64)	46 (64)
APL	1 (6)	1 (2)	2 (3)
First diagnosis or recurrent disease			
First diagnosis	12 (71)	46 (84)	58 (81)
Relapsed disease	5 (29)	9 (16)	14 (19)
Treatment phase			
Remission induction	13 (77)	47 (86)	60 (83)
Consolidation therapy	1 (6)	1 (2)	2 (3)
Allogeneic SCT	1 (6)	1 (2)	2 (3)
Other	2 (12)	6 (11)	8 (11)
Non-matching variables			
Sex			
Female	8 (47)	21 (38)	29 (40)
Male	9 (53)	34 (62)	43 (60)
Age^§^	65 (52 to 70)	57 (42 to 68)	58 (43 to 68.5)
Death†	8 (47)	5 (9)	13 (18)

*Values are numbers (percentage of total) unless specified differently

^§^Age in years, median (IQR)

^†^Mortality not specific to bleeding (all-cause mortality)

Since controls are matched to cases, numbers presented for controls are dependent on control selection and therefore cannot be compared with numbers presented for cases

*ALL*, acute lymphoid leukemia; *AML*, acute myeloid leukemia; *MDS*, myelodysplastic syndrome; *APL*, acute promyelocytic leukemia; *SCT*, stem cell transplantation

In total, 482 platelet count tests were performed for all cases and controls in the implicated seven-day periods; of these, 56 (11.6%) were ≤ 10 × 10^9^/L (from a total of 27 of 72 included patients) and 138 (28.6%) were ≤ 20 × 10^9^/L (from a total of 43 of 72 included patients). Numbers of cases and controls with low platelet counts per implicated periods are given in Table 2.

**Table 2:** Presence of low platelet count measures in case-control population and rate ratios for associations between three different measures for low platelet count and the incidence of intracranial hemorrhage during the next one, three, five and seven days among patients with acute leukemia

Platelet count	Implicated time period	Cases *n* = 17‡	Controls *n* = 55‡	RR (95% CI)
One or more platelet counts ≤ 10 × 10^9^/L	1 day	1 (6%)	5 (9%)	0.67 (0.06 to 7.00)
	3 days	6 (35%)	12 (22%)	1.94 (0.44 to 8.56)
	5 days	7 (41%)	17 (31%)	1.66 (0.41 to 6.79)
	7 days	8 (47%)	19 (35%)	1.79 (0.50 to 6.39)
One or more platelet counts ≤ 20 × 10^9^/L	1 day	8 (47%)	13 (24%)	3.64 (0.91 to 14.58)
	3 days	10 (59%)	22 (40%)	2.33 (0.63 to 8.62)
	5 days	13 (76%)	27 (49%)	5.47 (1.08 to 27.75)
	7 days	13 (76%)	30 (55%)	4.21 (0.83 to 21.26)
Percentage of hours platelet count ≤ 20 × 10^9^/L	1 day	6% (0% to 78%)	0% (0% to 100%)	1.01 (0.21 to 4.88)
	3 days	38% (0% to 51%)	0% (0% to 69%)	0.86 (0.16 to 4.47)
	5 days	28% (3% to 36%)	0% (0% to 43%)	1.90 (0.34 to 10.79)
	7 days	22% (2% to 33%)	4% (0% to 41%)	1.86 (0.30 to 11.57)

‡For one or more platelet counts ≤ 10 × 10^9^/L or ≤ 20 × 10^9^/L, the numbers represent the number of distinct cases or controls with platelet count measurements below 10 and 20 and the percentage according to the total of cases or controls. For percentage of hours platelet count ≤ 20 × 10^9^/L, the numbers represent the median and interquartile range

One or more platelet counts ≤ 10 × 10^9^/L: measure that describes the presence of at least one platelet count ≤ 10 × 10^9^/L within every defined implicated time period

One or more platelet counts ≤ 20 × 10^9^/L: measure that describes the presence of at least one platelet count ≤ 20 × 10^9^/L within every defined implicated time period

Percentage of hours platelet count ≤ 20 × 10^9^/L: the percentage of the number of hours that platelet count was ≤ 20 × 10^9^/L from the total number of hours between the first and last measurement of platelet count in each implicated time period. To calculate the number of hours with a platelet count ≤ 20 × 10^9^/L, a linear trend within two actual measurements was assumed and for every hour, the expected platelet count was interpolated. The percentage of hours with a platelet count ≤ 20 × 10^9^/L is a measure that describes duration of thrombocytopenia

Presented RRs are for a person with 100% of hours ≤ 20 × 10^9^/L, compared with 0% of hours. In the 7-day period, for a patient with 25% of hours with a platelet count ≤ 20 × 10^9L^/, the RR would be 1.86^0.25^ = 1.17; for a patient with 50% of hours with a platelet count ≤ 20 × 10^9^/L, the RR would be 1.86^0.50^ = 1.36; for 75% of hours ≤ 20 × 10^9^/L, it would be 1.86^0.75^ = 1.59, etc.

The median number of platelet transfusions per implicated period is presented in Table 3. For the seven-day period, cases had a median of three transfusions (range 0 to 12) and controls, a median of one transfusion (range 0 to 9). Other platelet product characteristics are presented in the supplementary material (Table S1). In total, case patients received 95 platelet transfusions, and control patients 107. Besides a higher total percentage of irradiated platelet products in the case patients (51.6% versus 38.3% in control patients), platelet product characteristics did not differ relevantly between cases and controls.

**Table 3:** Crude and adjusted rate ratios for the association between platelet transfusions and the incidence of intracranial hemorrhage among patients with acute leukemia

Implicated time period	Number of platelet transfusions*		Platelet transfusions	RR (95% CI)	
	Cases (*n* = 17)	Controls (*n* = 55)	Category	Crude	Adjusted for one or more platelet counts ≤ 10 × 10^9^/L
1 day	0 (0 to 2)	0 (0 to 2)	0	Ref	Ref
			1 to 2	3.86 (1.08 to 13.79)	4.50 (1.20 to 16.90)
			> 2	-	-
3 days	2 (0 to 4)	0 (0 to 5)	0	Ref	Ref
			1 to 2	2.32 (0.60 to 9.01)	2.36 (0.54 to 10.40)
			> 2	8.12 (0.80 to 82.2)	8.27 (0.73 to 93.51)
5 days	3 (0 to 9)	1 (0 to 7)	0	Ref	Ref
			1 to 2	2.16 (0.37 to 12.55)	2.21 (0.32 to 15.23)
			> 2	13.11 (1.91 to 90.03)	13.36 (1.78 to 100.28)
7 days	3 (0 to 12)	1 (0 to 9)	0	Ref	Ref
			1 to 2	4.04 (0.73 to 22.27)	4.09 (0.70 to 23.85)
			> 2	8.91 (1.53 to 51.73)	9.02 (1.47 to 55.49)

*Platelet transfusions, median (range): number of platelet transfusions received by case and control patients per implicated period

### Platelet count and the incidence of intracranial hemorrhage

To assess the impact of thrombocytopenia on intracranial hemorrhage for the four implicated time periods, we correlated our three defined measures of platelet count with the incidence of intracranial hemorrhage (Table 2).

When thrombocytopenia was defined as one or more count ≤ 10 × 10^9^/L, we observed that during the three, five and seven-day periods, the incidence of intracranial hemorrhage was higher after occurrence of such low platelet counts. For one or more count ≤ 20 × 10^9^/L, the association was present in all implicated periods. However, the confidence intervals are mostly very wide, compatible with the possibility of the true association showing both higher and lower incidences. When we assessed the association between the occurrence of one or more platelet count below 10 × 10^9^/L and intracranial hemorrhage in the seven-day period, we found an incidence rate ratio (RR) of 1.79 (95% confidence interval (CI) 0.50 to 6.39). In case of one or more platelet counts below 20 × 10^9^/L during the seven-day period, the RR was 4.21 (CI 0.83 to 21.26), meaning that patients with at least one platelet count below 20 × 10^9^/L most likely had a 4.21 higher rate of intracranial hemorrhage compared with patients with no platelet counts below 20 × 10^9^/L. For all the other time periods, RRs and CIs are given in Table 2.

Low platelet counts often lead to transfusion, meaning that the occurrence of low trough levels as assessed above does not take the precise time of deep thrombocytopenia into account. To assess the impact of the amount of time with thrombocytopenia, we next assessed the association between the percentage of hours with a platelet count ≤ 20 × 10^9^/L with the occurrence of intracranial hemorrhage. Since platelet count was not determined every hour, the percentage of hours with a platelet count ≤ 20 × 10^9^/L was calculated after interpolation of truly measured platelet counts leading to an estimated measure per hour (see Fig. 2). For the seven-day period, patients with 100% of hours at a platelet count ≤ 20 × 10^9^/L had a 1.86 (CI 0.30 to 11.57) higher rate of intracranial hemorrhage (reference 0%). This is the RR for 100% of the hours; for smaller percentages of hours, this RR can be calculated. For example, for a patient with 25% of hours at a platelet count ≤ 20 × 10^9^/L, the RR would be 1.86^0.25^ = 1.17. RRs for the other implicated periods for all three measures of a platelet count are shown in Table 2.

Most studies investigating bleeding risk in hemato-oncology patients take only patients receiving active treatment into account, not also patients who are admitted for treatment or disease-related complications. We did include the latter patient population, and to see if this affected our results, a post hoc sensitivity analysis excluding patients with other indications for admission then chemotherapy or stem cell transplantation was performed. This did not relevantly change the RRs for platelet count in intracranial hemorrhage (online supplementary material: Table S2).

Since there are potential confounding clinical factors that can influence the association of platelet count with intracranial hemorrhage, as predefined additional analysis, we corrected all analysis above for these variables that were collected from the electronic patient files. Table S3 (online supplementary material) presents this corrected RRs for the association of the differently defined measures of platelet count with the incidence of intracranial hemorrhage. Overall, results did not differ relevantly and/or consistently over the time periods.

### Platelet transfusions and the incidence of intracranial hemorrhage

Our findings indicated that the incidence of intracranial hemorrhage was higher in patients who had received platelet transfusions (Table 3). The RRs for 1–2 platelet transfusions compared with 0 were between 2.16 (CI 0.37 to 12.55) and 4.04 (CI 0.73 to 22.27) for the different implicated periods. The latter, for example, is the RR for the seven-day implicated period, indicating that the most likely incidence of intracranial hemorrhage for a patient who received 1 or 2 transfusions was 4.04 higher compared with a patient without platelet transfusions. For patients who received > 2 platelet transfusions, RRs differed between 8.12 (CI 0.80 to 82.20) and 13.11 (CI 1.91 to 90.03) for the different implicated periods, so the incidence of intracranial hemorrhage was up to 13.11 times as high in patients who received more than two transfusions compared with none.

Platelet transfusions are given in case of low platelet counts; therefore, they might be seen as a surrogate marker for thrombocytopenia. To assess if associations between platelet transfusion and intracranial hemorrhage were also independent of platelet counts, we adjusted for our defined measures of platelet count. The risk of increasing numbers of platelet transfusions on intracranial hemorrhage mostly stayed stable or increased in the case of one or more platelet counts ≤ 10 × 10^9^/L and percentage of hours with a platelet count ≤ 20 × 10^9^/L. RRs decreased in the case of one or more platelet counts ≤ 20 × 10^9^/L, but the direction of the effect stayed the same (one or more platelet counts ≤ 10 × 10^9^/L: see Table 3, other measures of platelet count: see online supplementary material, Table S4). As an additional explorative and predefined analysis, we assessed if the association was similar when looking at the number of transfused platelets on a continuous scale, instead of the categorical scale. For all investigated implicated periods, the incidence rates of intracranial hemorrhage were higher with an increasing number of units of transfused platelets (see online supplementary material: Table S5). The RRs ranged between 1.48 (CI 1.06 to 2.07) and 2.46 (CI 1.02 to 5.91) within the periods. These RRs are for one additional transfusion and increase rapidly if more transfusions are given. To illustrate, the crude RR for the 7-day period of 1.48 was for one additional platelet transfusion, if a patient had 2 platelet transfusions, the rate ratio would be 1.48^2^ = 2.19, for 3 transfusions 1.48^3^ = 3.24, etc.

Finally, since we expected that clinical conditions might influence the found associations, we also explored if the RRs for the association between platelet transfusion and intracranial hemorrhage were affected by potential confounders (see online supplementary material: Table S6). Adjustment for some clinical variables did decrease or increase the incidence rate ratio in a potentially relevant manner, which showed consistent directions within implicated periods. This means that the variables, fever, presence of a trauma like a fall or procedure, presence of non-intracranial bleedings, and usage of antiplatelet or anticoagulant medication, were potentially relevant confounding variables based on our data.

## Discussion

In this case-control study among leukemia patients, we observed that one or more platelet counts below thresholds of both 10 × 109/L and 20 × 10^9^/L, and an increasing percentage of hours below 20 × 10^9^/L was associated with intracranial hemorrhage, especially when low platelet counts occurred more than one day before the event of the hemorrhage. However, the estimates of these associations lacked precision. Platelet transfusions were also associated with the occurrence of subsequent intracranial hemorrhage; these estimates of association were likewise imprecise.

The point estimates of the association between all the defined measures of low platelet counts and the incidence of intracranial hemorrhage show a clear trend of higher incidences of intracranial hemorrhage when platelet counts are low. The most likely rate ratios are especially increased if platelet counts were low at three, five or seven days before the hemorrhage. In contrast, no increased incidence is seen in the period of 1 day before hemorrhage for two out of our three defined measures of platelet count. Although almost all point estimates go in the same direction, and an increased incidence of intracranial hemorrhage when platelet counts are low is thus most likely, the confidence intervals are wide, due to low numbers of patients. This means that the true effect size could lay in a wide range of values, from strongly harmful to even protective.

Quantitative evidence on the association between platelet counts and the occurrence of intracranial hemorrhage among patients with leukemia is scarce. Some reports focused on fatal intracranial hemorrhage [2, 5–7, 15]. One study did find an association between thrombocytopenia and the occurrence of intracranial hemorrhage in a subgroup of post-allogeneic stem cell transplantation patients [12]. Two RCTs investigated the effect of prophylactic platelet transfusions on the occurrence of bleeding. Therapeutically treated patients had lower platelet counts compared with prophylactically transfused patients. One RCT did not find a difference in the occurrence of grade 3 and 4 bleedings (including intracranial hemorrhage) [26] while the other did see more intracerebral hemorrhage in the therapeutically transfused group [16]. However, the latter RCT had a different CT scan policy for both study arms, which likely reduced the number of confirmed intracranial hemorrhage in the control arm.

Moreover, most studies describe associations of bleeding with platelet counts of only one day or do not clarify fully which platelet counts are taken into consideration for the analysis. However, it has also been suggested that there may be a longer lag time before low platelet counts can lead to bleeding [18]. Our results suggest that potentially prolonged thrombocytopenia (3 to 7 days) is leading to more intracranial hemorrhages. Our study is as far as we know the first to define several implicated periods and several measures of platelet count, to investigate the association between both time and trough of low platelet counts and intracranial hemorrhage.

Platelet counts are not surprisingly strongly related to platelet transfusions in this patient population. Low platelet counts lead to transfusions, and transfusions affect future platelet counts. Since in this study we also saw an association between platelet transfusions and intracranial hemorrhage, ideally you would like to adjust for the potential confounding effect of platelet transfusions. However, this is extremely difficult, even if one would have a large dataset, given that platelet counts and platelet transfusions are so strongly interdependent, and that multiple platelet counts, and transfusions would need to be considered (see online supplementary material: fig. S1). In our small sample size, such corrections are impossible.

In the present study, also platelet transfusions were associated with an increased incidence of intracranial hemorrhage, especially when > 2 transfusions were given in an implicated period.

Since low platelet counts are often the reason for platelet transfusion, we aimed to correct for the defined measures of platelet count. Due to the fact that patients often had multiple transfusions and multiple platelet count determinations, a reliable and complete correction is again not possible in our dataset. Nevertheless, by adding the different defined measures of platelet count into the model, we see that this did not influence the observed association between platelet transfusion and intracranial hemorrhage in our study. Therefore, we infer that it seems plausible that the need for platelet transfusions or platelet transfusions itself in the circumstances where they are frequently needed might increase the incidence of intracranial hemorrhage and that this is at least partly independent of platelet counts. However, other clinical factors that lead to an increasing need for platelet transfusions, for example, conditions leading to increased platelet consumption, are very likely responsible for the latter observed association with intracranial hemorrhage. To investigate the impact of such potential confounding clinical conditions, we corrected them by adding relevant clinical factors in the regression model. Indeed, we identified anticoagulation/antiplatelet therapy and other (non-intracranial) bleeding events as possible confounders. These were also previously suggested to increase bleeding risk in hemato-oncology patients [22]. For causal interpretation, an extensive multivariable model in an individual patient data meta-analysis of studies like ours would be essential allowing adjustment for all confounding. Besides confounding, the observed association between platelet transfusions and intracranial hemorrhage may also be due to relative functional defects of the transfused platelets. Platelet concentrates are known to develop storage lesions, which can lead to reduced platelet quality [33, 34]. Moreover, one could argue that the transfusions contribute to intracranial hemorrhage by other mechanisms. Platelets do not only act in primary hemostasis but also have immunomodulatory functions. Inflammation is likely to influence bleeding risks, especially in thrombocytopenic conditions [17, 35–39]. The idea that platelet transfusions lead to adverse outcomes is indeed reported by two RCTs, both showing adverse effects on morbidity and mortality in very different patients’ populations, namely patients with a hemorrhagic cerebral vascular accident while using antiplatelet agents and thrombocytopenic neonates [28, 29]. The mechanisms behind these findings, however, are unclear. Finally, the observed associations could also be due to chance.

### Strengths and limitations

A strength of this study is the matching of case and control patients on diagnosis and treatment. This allowed adjustment for these important known risk factors for this rare, but feared, bleeding complication.

Also, matching on the hospital was performed to correct for confounders that are not easily quantified, like differences in local treatment policies. Additionally, we matched case and control patients on time after starting a treatment or after the admission date. During admission, a leukemia patient is likely exposed to different platelet counts and other clinical risk factors, mostly determined by the exposure to intensive cytoreductive treatment. By matching case and control patients on time after therapy/admission, we minimized confounding by direct treatment effects.

Another asset of the study is the completeness of information for our main variables, namely platelet counts and platelet transfusions. A strong feature of the study is that we examined multiple measures of platelet counts during a week before the intracranial hemorrhage. With these different measures, we could explore various possible influences of thrombocytopenia, like trough level and duration, on the incidence of intracranial hemorrhage during one, three, five and seven days before the hemorrhage. To the best of our knowledge, this has not been performed in other studies.

Finally, our study may be a novel framework which enables taking time aspects and thrombocytopenia severity into account. Our nested case-control study, that to our knowledge was not applied before, allowed exploration of effects of time and severity, via defining various implicated time periods for multiple measures of the exposure on the outcome intracranial hemorrhage.

Our study has also some limitations. First, our sample size was too small to assess some potentially interesting and relevant measures of platelet count. Since patients are transfused as soon as platelet counts drop below 10 × 10^9^/L, the time below this value could not be sufficiently assessed. Even with a larger study population, the frequent transfusions would likely still minimize the amount of time ≤ 10 × 10^9^/L. Therefore, although this cutoff point is the most widely used transfusion trigger, we could not assess the effect of time below 10 × 10^9^/L on the occurrence of intracranial hemorrhage.

Furthermore, as discussed earlier, due to the small sample size, we could only correct for one variable at the time. Therefore, by the lack of multivariate analysis, residual confounding remains. While we aimed to assess causality, although proving causality is never possible [40, 41], all results have to be interpreted as hypothesis generating only. Confirmation in larger studies will be necessary, although challenging due to the rarity of intracranial hemorrhage. In addition, biological mechanisms should be investigated.

Also, we may have missed patients that acutely died due to severe intracranial hemorrhage, leading to potential bias. These patients remain undetected in the applied algorithm due to the absence of laboratory or additional diagnostics. The number of these missed patients is likely to be very limited. So, a relevant change of the findings is not to be expected, except for inducing a lower incidence of intracranial hemorrhage. Finally, given the retrospective nature of collecting data, it was not always possible to distinguish if platelet transfusions were truly prophylactic. Transfusion triggers were often not recorded clearly and might have been higher than 10 × 10^9^/L in case of an assumed higher bleeding risk [22–24]. Possibly also some therapeutic transfusions might have been included if they were actually given for an unrecorded (probably minor) bleeding event. Patients who already need therapeutically platelet transfusions have proven to be more prone to bleeding and thereby are likely to also have a higher risk for intracranial bleeding.

## Conclusion

In summary, we quantified the association between low platelet counts and the incidence of intracranial hemorrhage in leukemia patients. Longer periods of thrombocytopenia were associated with a higher risk.

The number of administered platelet transfusions was also associated with intracranial hemorrhage. Incidences especially increased for patients receiving > 2 platelet transfusions. Nonetheless, this study cannot imply any causality between the platelet transfusion and intracranial hemorrhage. More likely, our findings suggest that there is an association between platelet transfusion and other clinical risk factors that lead to an increased transfusion need. Indeed, this observed association should not lead to withholding prophylactic platelet transfusions. Future research needs to establish whether and when platelet transfusions or other possible preventive measures provide protection against intracranial hemorrhage among patients with leukemia or not.

## Electronic supplementary material

ESM 1 (DOCX 278 kb)

## Data Availability

Data and codes are accessible by the data management team. On request, after approval of the last author and if a legal data sharing agreement is arranged, data and/or codes might be transferred, without any identifying information of subjects.

## References

[1] Ypma PF, Kerkhoffs JL, van Hilten JA, Middelburg RA, Coccoris M, Zwaginga JJ, Beckers EM, Fijnheer R, van der Meer PF, Brand A (2012). The observation of bleeding complications in haemato-oncological patients: stringent watching, relevant reporting. Transfus Med.

[2] Chen CY, Tai CH, Cheng A, Wu HC, Tsay W, Liu JH, Chen PY, Huang SY, Yao M, Tang JL, Tien HF (2012). Intracranial hemorrhage in adult patients with hematological malignancies. BMC Med.

[3] Chern JJ, Tsung AJ, Humphries W, Sawaya R, Lang FF (2011). Clinical outcome of leukemia patients with intracranial hemorrhage. Clinical article. J Neurosurg.

[4] Groch SN, Sayre GP, Heck FJ (1960). Cerebral hemorrhage in leukemia. Arch Neurol.

[5] Chen CY, Tai CH, Tsay W, Chen PY, Tien HF (2009). Prediction of fatal intracranial hemorrhage in patients with acute myeloid leukemia. Ann Oncol.

[6] Dayyani F, Mougalian SS, Naqvi K, Shan J, Ravandi F, Cortes J, Weinberg J, Jabbour E, Faderl S, Wierda W, Thomas D, O’Brien S, Pierce S, Kantarjian H, Garcia-Manero G (2011). Prediction model for mortality after intracranial hemorrhage in patients with leukemia. Am J Hematol.

[7] Kim H, Lee JH, Choi SJ, Lee JH, Seol M, Lee YS, Kim WK, Lee JS, Lee KH (2006). Risk score model for fatal intracranial hemorrhage in acute leukemia. Leukemia.

[8] Ariesen MJ, Claus SP, Rinkel GJ, Algra A (2003). Risk factors for intracerebral hemorrhage in the general population: a systematic review. Stroke.

[9] Sturgeon JD, Folsom AR, Longstreth WT, Shahar E, Rosamond WD, Cushman M (2007). Risk factors for intracerebral hemorrhage in a pooled prospective study. Stroke.

[10] An SJ, Kim TJ, Yoon BW (2017). Epidemiology, risk factors, and clinical features of intracerebral hemorrhage: an update. J Stroke.

[11] Rogers LR (2010). Cerebrovascular complications in patients with cancer. Semin Neurol.

[12] Zhang XH, Wang QM, Chen H, Chen YH, Han W, Wang FR, Wang JZ, Zhang YY, Mo XD, Chen Y, Wang Y, Chang YJ, Xu LP, Liu KY, Huang XJ (2016). Clinical characteristics and risk factors of intracranial hemorrhage in patients following allogeneic hematopoietic stem cell transplantation. Ann Hematol.

[13] Najima Y, Ohashi K, Miyazawa M, Nakano M, Kobayashi T, Yamashita T, Akiyama H, Sakamaki H (2009). Intracranial hemorrhage following allogeneic hematopoietic stem cell transplantation. Am J Hematol.

[14] Velander AJ, DeAngelis LM, Navi BB (2012). Intracranial hemorrhage in patients with cancer. Curr Atheroscler Rep.

[15] Zhang Q, Li X, Wei Z, Ye X, Zhu L, Xie M, Xie W, Zhu J, Li L, Zhou ZY, Yang X, Zhu M, Sun J (2017). Risk factors and clinical characteristics of non-promyelocytic acute myeloid leukemia of intracerebral hemorrhage: a single center study in China. J Clin Neurosci.

[16] Wandt H, Schaefer-Eckart K, Wendelin K, Pilz B, Wilhelm M, Thalheimer M, Mahlknecht U, Ho A, Schaich M, Kramer M, Kaufmann M, Leimer L, Schwerdtfeger R, Conradi R, Dolken G, Klenner A, Hanel M, Herbst R, Junghanss C, Ehninger G, Study Alliance L (2012). Therapeutic platelet transfusion versus routine prophylactic transfusion in patients with haematological malignancies: an open-label, multicentre, randomised study. Lancet.

[17] Stanworth SJ, Hudson CL, Estcourt LJ, Johnson RJ, Wood EM, Invest TS (2015). Risk of bleeding and use of platelet transfusions in patients with hematologic malignancies: recurrent event analysis. Haematologica.

[18] Middelburg RA, Kerkhoffs JH, van der Bom JG (2018). Thrombocytopenia and bleeding in myelosuppressed transfusion-dependent patients: a simulation study exploring underlying mechanisms. Clin Epidemiol.

[19] Estcourt LJ, Birchall J, Lowe D, Grant-Casey J, Rowley M, Murphy MF (2012). Platelet transfusions in haematology patients: are we using them appropriately?. Vox Sang.

[20] Kumar A, Mhaskar R, Grossman BJ, Kaufman RM, Tobian AA, Kleinman S, Gernsheimer T, Tinmouth AT, Djulbegovic B, Panel APTG (2015) Platelet transfusion: a systematic review of the clinical evidence. Transfusion 55 (5):1116–1127; quiz 1115. 10.1111/trf.12943 10.1111/trf.12943 25387589

[21] Charlton A, Wallis J, Robertson J, Watson D, Iqbal A, Tinegate H (2014). Where did platelets go in 2012? A survey of platelet transfusion practice in the North of England. Transfusion Med.

[22] Estcourt LJ, Birchall J, Allard S, Bassey SJ, Hersey P, Kerr JP, Mumford AD, Stanworth SJ, Tinegate H, Haematology BCS (2017). Guidelines for the use of platelet transfusions. Brit J Haematol.

[23] Schiffer CA, Bohlke K, Delaney M, Hume H, Magdalinski AJ, McCullough JJ, Omel JL, Rainey JM, Rebulla P, Rowley SD, Troner MB, Anderson KC (2018). Platelet transfusion for patients with cancer: American Society of Clinical Oncology clinical practice guideline update. J Clin Oncol.

[24] de Vries R, Haas F, working group for revision of the Dutch Blood Transfusion G (2012). English translation of the Dutch blood transfusion guideline 2011. Clin Chem.

[25] Miller AB, Hoogstraten B, Staquet M, Winkler A (1981). Reporting results of cancer treatment. Cancer.

[26] Stanworth SJ, Estcourt LJ, Powter G, Kahan BC, Dyer C, Choo L, Bakrania L, Llewelyn C, Littlewood T, Soutar R, Norfolk D, Copplestone A, Smith N, Kerr P, Jones G, Raj K, Westerman DA, Szer J, Jackson N, Bardy PG, Plews D, Lyons S, Bielby L, Wood EM, Murphy MF, Investigators T (2013). A no-prophylaxis platelet-transfusion strategy for hematologic cancers. N Engl J Med.

[27] Stanworth SJ, Estcourt LJ, Llewelyn CA, Murphy MF, Wood EM, Investigators TS (2014). Impact of prophylactic platelet transfusions on bleeding events in patients with hematologic malignancies: a subgroup analysis of a randomized trial. Transfusion.

[28] Baharoglu MI, Cordonnier C, Al-Shahi Salman R, de Gans K, Koopman MM, Brand A, Majoie CB, Beenen LF, Marquering HA, Vermeulen M, Nederkoorn PJ, de Haan RJ, Roos YB, Investigators P (2016). Platelet transfusion versus standard care after acute stroke due to spontaneous cerebral haemorrhage associated with antiplatelet therapy (PATCH): a randomised, open-label, phase 3 trial. Lancet.

[29] Curley A, Stanworth SJ, Willoughby K, Fustolo-Gunnink SF, Venkatesh V, Hudson C, Deary A, Hodge R, Hopkins V, Lopez Santamaria B, Mora A, Llewelyn C, D’Amore A, Khan R, Onland W, Lopriore E, Fijnvandraat K, New H, Clarke P, Watts T, PlaNe TMC (2019). Randomized trial of platelet-transfusion thresholds in neonates. N Engl J Med.

[30] Kreuger AL, Middelburg RA, Beckers EAM, de Vooght KMK, Zwaginga JJ, Kerkhoffs JH, van der Bom JG (2018). The identification of cases of major hemorrhage during hospitalization in patients with acute leukemia using routinely recorded healthcare data. PLoS One.

[31] Grimes DA, Schulz KF (2005). Compared to what? Finding controls for case-control studies. Lancet.

[32] Miettinen O (1976). Estimability and estimation in case-referent studies. Am J Epidemiol.

[33] Shrivastava M (2009). The platelet storage lesion. Transfus Apher Sci.

[34] Middelburg RA, Roest M, Ham J, Coccoris M, Zwaginga JJ, van der Meer PF (2013). Flow cytometric assessment of agonist-induced P-selectin expression as a measure of platelet quality in stored platelet concentrates. Transfusion.

[35] Webert K, Cook RJ, Sigouin CS, Rebulla P, Heddle NM (2006). The risk of bleeding in thrombocytopenic patients with acute myeloid leukemia. Haematologica.

[36] Friedmann AM, Sengul H, Lehmann H, Schwartz C, Goodman S (2002). Do basic laboratory tests or clinical observations predict bleeding in thrombocytopenic oncology patients? A reevaluation of prophylactic platelet transfusions. Transfus Med Rev.

[37] Stolla M, Refaai MA, Heal JM, Spinelli SL, Garraud O, Phipps RP, Blumberg N (2015). Platelet transfusion - the new immunology of an old therapy. Front Immunol.

[38] Keller TT, Mairuhu AT, de Kruif MD, Klein SK, Gerdes VE, ten Cate H, Brandjes DP, Levi M, van Gorp EC (2003). Infections and endothelial cells. Cardiovasc Res.

[39] Goerge T, Ho-Tin-Noe B, Carbo C, Benarafa C, Remold-O’Donnell E, Zhao BQ, Cifuni SM, Wagner DD (2008). Inflammation induces hemorrhage in thrombocytopenia. Blood.

[40] Hernan M (2018). The C-word: the more we discuss it, the less dirty it sounds. Am J Public Health.

[41] Hernan MA (2018). The C-word: scientific euphemisms do not improve causal inference from observational data. Am J Public Health.

