# Supplementary material for: Thrombocytopenia and the effect of platelet transfusions on the occurrence of intracranial hemorrhage in patients with acute leukemia – a nested case-control study
Source: Ann Hematol. 2020 Oct 17;100(1):261–71. doi: 10.1007/s00277-020-04298-7 (PMC7782440; doi:10.1007/s00277-020-04298-7)
Supplement: Supplementary file 1 — (DOCX 278 kb) [file 277_2020_4298_MOESM1_ESM.docx]

**Supplementary material**

**Title**: Thrombocytopenia and the effect of platelet transfusions on the occurrence of intracranial hemorrhage in patients with acute leukemia – a nested case control study

**Authors**: Cornelissen LL; Kreuger AL; Caram-Deelder C; Middelburg RA; Kerkhoffs JLH; Zwaginga JJ; von dem Borne PA; Beckers EAM; de Vooght KMK; Kuball J; van der Bom JG (corresponding autohor; j.g.van_der_bom@lumc.nl)

**Journal**: submitted to Annals of Hematology

**DOI**: follows publication

**Table of contents**

[Data collection 2](#_Toc51749118)

[Figure S1. Interdependence of platelet count and platelet transfusion 3](#_Toc51749119)

[Table S1. Distribution of platelet product characteristics 4](#_Toc51749120)

[Table S2. Crude rate ratios (RR) for platelet count on ICH in total population of patients with all indications for admission, and in sub-population of patients who are admitted for chemotherapy or stem cell transplantation 5](#_Toc51749121)

[Table S3. Crude rate ratios (RR) for platelet count on ICH and adjusted RR’s for clinical risk factors (n=72). 6](#_Toc51749122)

[Table S4. Crude and adjusted rate ratios for the association between platelet transfusions and the incidence of intracranial hemorrhage among patients with acute leukemia 13](#_Toc51749123)

[Table S5. Crude and adjusted rate ratios*, for the association between platelet transfusions (on a continuous scale) and the incidence of intracranial hemorrhage among patients with acute leukemia 14](#_Toc51749124)

[Table S6. Crude rate ratios (RR) for platelet (PLT) transfusions on ICH and adjusted RR’s for clinical risk factors. 15](#_Toc51749125)

# Data collection

Laboratory and transfusion data were extracted from medical records electronically. Laboratory data consisted of all measured platelet counts, leukocyte counts and hemoglobin levels. Transfusion data consisted of all given platelet transfusions and red blood cell transfusions. Platelet products that were used were standard 5-donor buffy coat derived concentrates that were leuko-depleted. If there was a clinical indication, the product could be ABO matched, HLA matched or radiated.

Clinical data of the included case and control patients were collected by manual chart review. Data were entered using a secure online Case Report Form. All variables that could change over time were collected for every separate day in the week preceding the index date. Variables collected were:

- General characteristics: age, WHO performance score at admission, BMI, intoxications (alcohol and smoking), ABO blood group, hospital
- Bleeding characteristics (cases only): bleeding description, interventions after bleeding, date of clinically relevant bleeding (= index day, NB: controls also have a corresponding index day registered)
- Data about diagnosis and indication for admission: Diagnosis in groups, as well as described in conclusions, disease activity at index day (active disease, partial remission, complete remission), disease status (new, relapse, transformation etc.), indication for admission (e.g. remission induction chemotherapy, consolidation therapy, allogeneic stem cell transplantation (SCT), etc., all including description), transplantation details, date of start of treatment/admission
- Comorbidities: description of present comorbidities, need for usage of antihypertensive medication/ cholesterol-lowering medication/medication for diabetes mellitus/medication for ischemic heart disease, bleeding events reported in medical history before diagnosis, presence of graft versus host disease in index period
- Medication in 10 days before index day: e.g. anti-coagulant medication, antiplatelet medication, anti-infectious medication, chemotherapy, immuno-suppressive medication, etc.
- Infection data in 7 days before index day: highest temperature per day (in case temperature was higher than 38 degrees Celsius), presence or suspicion of infection, results of cultures and PCR’s, active infection treatment, radiology results, infection in conclusion or differential diagnosis, etc.
- Non-intracranial bleedings in 7 days before index day: presence described in medical records, and if so, description and WHO bleeding grade.
- Transfusion data in 7 days before index day: Triggers for platelet and erythrocyte transfusions, prophylactic or therapeutic transfusions, number of transfused products, platelet refractoriness described
- Other possible risk factors in 7 days before index day: presence of trauma or intervention (including lumbar punctures), vomiting

# Figure S1. Interdependence of platelet count and platelet transfusion

*
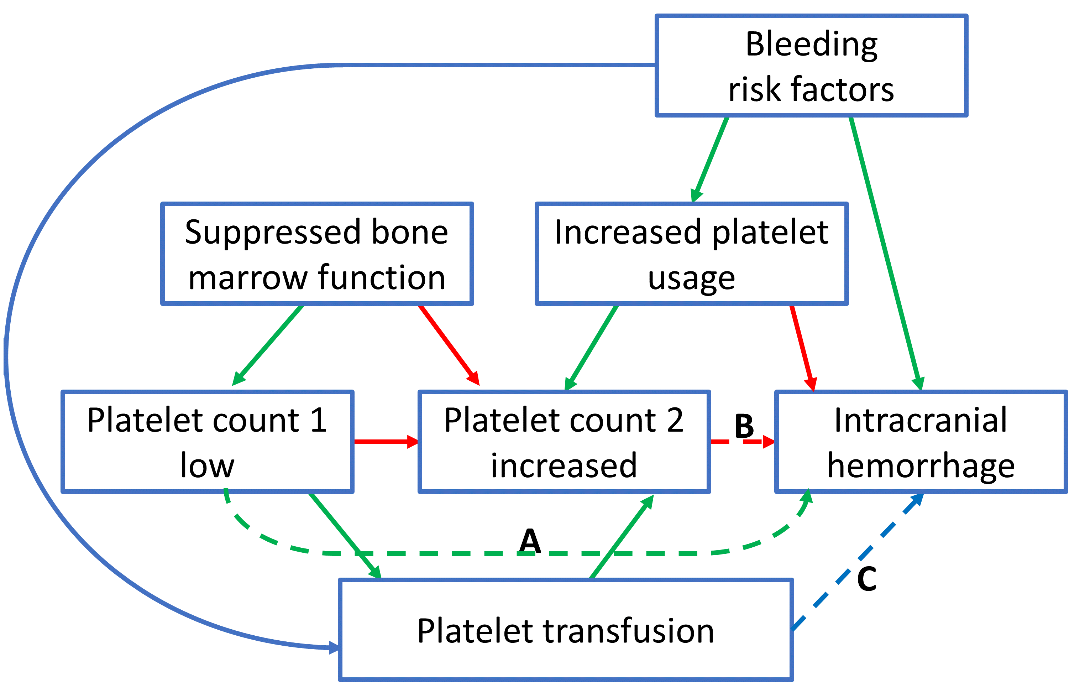
*

Platelet count and platelet transfusion are clearly interdependent, making adequate correction for each other very hard.

Dotted lines represent the studied associations. Green arrows are (likely) inducing effects, while red arrows are likely inhibitors.

The **A** and **B** represent the studied associations between low platelet count(s) and intracranial hemorrhage.

Ad **A**. Low platelet counts before transfusions, are lower because of suppressed marrow function and by usage of platelets e.g. in hemostasis. The low platelet counts however increase (green arrow A) the risk of intracranial hemorrhage. Due to platelet count triggers the low platelet count 1 leads to platelet transfusions, which increases the platelet counts after the transfusion(s).

Ad **B**. The deeper the through of the low platelet counts the more the effect of transfusions on platelet count increase is mitigated. However, more increased post transfusion platelet counts inhibit intracranial hemorrhage.

The **C** represents the observed positive association between platelet transfusion and intracranial hemorrhage. However, this association is not easily to evaluate apart from the association A between low platelet counts and hemorrhage, since low platelet counts increases the number of transfusions. Also, the number of platelet transfusions is dependent on transfusion triggers, that can be adjusted based on bleeding risk factors: the association of platelet transfusion with ICH might also be based on bleeding risk factors leading to increased transfusion needs/increased triggers.

# Table S1. Distribution of platelet product characteristics

| **Platelet product characteristics** | **Cases n=17**  **95 platelet transfusions** | **Controls n=55**  **107 platelet transfusions** | **Total n =72**  **202 platelet transfusions** |
| --- | --- | --- | --- |
| **Irradiation*** |  |  |  |
| Yes | 49 (51.6%) | 41 (38.3%) | 90 (44.6%) |
| No | 46 (48.4%) | 66 (61.7%) | 112 (55.5%) |
| **Number of donors*** |  |  |  |
| Apheresis – single donor | 3 (3.2%) | 9 (8.4%) | 12 (5.9%) |
| Pooled – five donors | 92 (96.8%) | 98 (91.6%) | 190 (94.1%) |
| **Hyperconcentrated*** |  |  |  |
| Yes | 13 (13.7%) | 11 (10.3%) | 24 (11.9%) |
| No | 82 (86.3%) | 96 (89.7%) | 178 (88.1%) |
| **Storage medium*** |  |  |  |
| Plasma | 73 (76.8%) | 90 (84.1%) | 163 (80.7%) |
| Pas-C | 22 (23.2%) | 17 (15.9%) | 39 (19.3%) |
| **Storage time (days)**§ | 4.3 (1.3) | 4.6 (1.6) | 4.4 (1.5) |

* Results are number (percentage)

§ Results are mean (standard deviation)

Individual patients could have received a combination of products for all categories.

#

# Table S2. Crude rate ratios (RR) for platelet count on ICH in total population of patients with all indications for admission, and in sub-population of patients who are admitted for chemotherapy or stem cell transplantation

|  |  | **RR (95% CI)** | | | |
| --- | --- | --- | --- | --- | --- |
| **Platelet count** | **Index period** | **Total**  **Population**  **n=72** | **Sub-group**  **n=64** |  |  |
| **Percentage of hours platelet count ≤20 x10^9^/L** | | | | | |
|  | 1 day | 1.01 (0.21 to 4.88) | 0.79 (0.15 to 4.16) |  |  |
|  | 3 days | 0.86 (0.16 to 4.47) | 0.71 (0.13 to 3.98) |  |  |
|  | 5 days | 1.90 (0.34 to 10.79) | 1.72 (0.30 to 10.00) |  |  |
|  | 7 days | 1.86 (0.30 to 11.57) | 1.66 (0.26 to 10.54) |  |  |
| **One or more platelet counts ≤20 x10^9^/L** | | | | | |
|  | 1 day | 3.64 (0.91 to 14.58) | 3.11 (0.75 to 12.90) |  |  |
|  | 3 days | 2.33 (0.63 to 8.62) | 1.92 (0.50 to 7.45) |  |  |
|  | 5 days | 5.47 (1.08 to 27.75) | 4.66 (0.88 to 24.54) |  |  |
|  | 7 days | 4.21 (0.83 to 21.26) | 3.46 (0.66 to 18.10) |  |  |
| **One or more platelet counts ≤10 x10^9^/L** | | | | | |
|  | 1 day | 0.67 (0.06 to 7.00) | 0.67 (0.06 to 7.00) |  |  |
|  | 3 days | 1.94 (0.44 to 8.56) | 1.94 (0.44 to 8.56) |  |  |
|  | 5 days | 1.66 (0.41 to 6.79) | 1.66 (0.41 to 6.79) |  |  |
|  | 7 days | 1.79 (0.50 to 6.39) | 1.79 (0.50 to 6.39) |  |  |

Post hoc sensitivity analysis, exclusion of all patients admitted for another indication then chemotherapy or stem cell therapy.

Total population cases n=17, controls n=55. Subgroup analysis cases n=15, controls n=49.

# Table S3. Crude rate ratios (RR) for platelet count on ICH and adjusted RR’s for clinical risk factors (n=72).

| **Measurement of platelet count** | **Index period** | **Crude** | **Adjusted for sex**  **female (male is ref)** |
| --- | --- | --- | --- |
| **Percentage of hours platelet count ≤20 x10^9^/L** | |  |  |
|  | 1 day | 1.01 (0.21 to 4.88) | 1.03 (0.21 to 5.04) |
|  | 3 days | 0.85 (0.16 to 4.47) | 0.96 (0.18 to 5.24) |
|  | 5 days | 1.90 (0.34 to 10.79) | 2.13 (0.36 to 12.68) |
|  | 7 days | 1.86 (0.30 to 11.56) | 1.99 (0.31 to 12.78) |
| **One or more platelet counts ≤20 x10^9^/L** | |  |  |
|  | 1 day | 3.64 (0.91 to 14.58) | 3.88 (0.93 to 16.15) |
|  | 3 days | 2.33 (0.63 to 8.62) | 2.41 (0.64 to 8.98) |
|  | 5 days | 5.47 (1.08 to 27.75) | 5.86 (1.12 to 30.61) |
|  | 7 days | 4.21 (0.83 to 21.26) | 4.36 (0.85 to 22.36) |
| **One or more platelet counts ≤10 x10^9^/L** | |  |  |
|  | 1 day | 0.67 (0.06 to 7.00) | 0.64 (0.06 to 6.59) |
|  | 3 days | 1.94 (0.44 to 8.56) | 2.02 (0.45 to 9.05) |
|  | 5 days | 1.66 (0.41 to 6.79) | 1.68 (0.41 to 6.87) |
|  | 7 days | 1.79 (0.50 to 6.39) | 1.87 (0.52 to 6.79) |
| **Measurement of platelet count** | **Index period** | **Crude** | **Adjusted for age  (per additional year)** |
| **Percentage of hours platelet count ≤20 x109/L** | |  |  |
|  | 1 day | 1.01 (0.21 to 4.88) | 1.73 (0.29 to 10.18) |
|  | 3 days | 0.85 (0.16 to 4.47) | 1.20 (0.19 to 7.38) |
|  | 5 days | 1.90 (0.34 to 10.79) | 2.95 (0.41 to 21.28) |
|  | 7 days | 1.86 (0.30 to 11.56) | 3.55 (0.40 to 31.27) |
| **One or more platelet counts ≤20 x10^9^/L** | |  |  |
|  | 1 day | 3.64 (0.91 to 14.58) | 3.61 (0.88 to 14.77) |
|  | 3 days | 2.33 (0.63 to 8.62) | 2.31 (0.62 to 8.68) |
|  | 5 days | 5.47 (1.08 to 27.75) | 6.56 (1.08 to 39.43) |
|  | 7 days | 4.21 (0.83 to 21.26) | 4.63 (0.85 to 25.25) |
| **One or more platelet counts ≤10 x109/L** | |  |  |
|  | 1 day | 0.67 (0.06 to 7.00) | 0.75 (0.07 to 7.66) |
|  | 3 days | 1.94 (0.44 to 8.56) | 1.78 (0.37 to 8.62) |
|  | 5 days | 1.66 (0.41 to 6.79) | 1.45 (0.32 to 6.47) |
|  | 7 days | 1.79 (0.50 to 6.39) | 1.97 (0.47 to 8.17) |
| **Measurement of platelet count** | **Index period** | **Crude** | **Adjusted for antiplatelet or therapeutic anticoagulation medication within 10 days (Yes/No)** |
| **Percentage of hours platelet count ≤20 x109/L** | |  |  |
|  | 1 day | 1.01 (0.21 to 4.88) | 0.88 (0.18 to 4.27) |
|  | 3 days | 0.85 (0.16 to 4.47) | 0.87 (0.16 to 4.77) |
|  | 5 days | 1.90 (0.34 to 10.79) | 1.96 (0.31 to 12.27) |
|  | 7 days | 1.86 (0.30 to 11.56) | 1.78 (0.27 to 11.68) |
| **One or more platelet counts ≤20 x10^9^/L** | |  |  |
|  | 1 day | 3.64 (0.91 to 14.58) | 3.53 (0.89 to 14.32) |
|  | 3 days | 2.33 (0.63 to 8.62) | 2.30 (0.55 to 9.61) |
|  | 5 days | 5.47 (1.08 to 27.75) | 4.34 (0.75 to 24.87) |
|  | 7 days | 4.21 (0.83 to 21.26) | 3.64 (0.67 to 19.70) |
| **One or more platelet counts ≤10 x109/L** | |  |  |
|  | 1 day | 0.67 (0.06 to 7.00) | 0.67 (0.06 to 7.00) |
|  | 3 days | 1.94 (0.44 to 8.56) | 1.94 (0.44 to 8.56) |
|  | 5 days | 1.66 (0.41 to 6.79) | 1.39 (0.32 to 6.06) |
|  | 7 days | 1.79 (0.50 to 6.39) | 1.87 (0.47 to 7.47) |
| **Measurement of platelet count** | **Index period** | **Crude** | **Adjusted for cardiovascular disease risk score (per additional risk factor)** |
| **Percentage of hours platelet count ≤20 x109/L** | |  |  |
|  | 1 day | 1.01 (0.21 to 4.88) | 1.80 (0.30 to 10.71) |
|  | 3 days | 0.85 (0.16 to 4.47) | 1.08 (0.20 to 5.88) |
|  | 5 days | 1.90 (0.34 to 10.79) | 2.53 (0.42 to 15.14) |
|  | 7 days | 1.86 (0.30 to 11.56) | 2.52 (0.36 to 17.25) |
| **One or more platelet counts ≤20 x10^9^/L** | |  |  |
|  | 1 day | 3.64 (0.91 to 14.58) | 4.07 (0.92 to 17.94) |
|  | 3 days | 2.33 (0.63 to 8.62) | 3.62 (0.81 to 16.27) |
|  | 5 days | 5.47 (1.08 to 27.75) | 7.59 (1.23 to 47.01) |
|  | 7 days | 4.21 (0.83 to 21.26) | 5.69 (0.99 to 32.74) |
| **One or more platelet counts ≤10 x109/L** | |  |  |
|  | 1 day | 0.67 (0.06 to 7.00) | 0.97 (0.09 to 10.26) |
|  | 3 days | 1.94 (0.44 to 8.56) | 2.11 (0.46 to 9.77) |
|  | 5 days | 1.66 (0.41 to 6.79) | 1.36 (0.33 to 5.58) |
|  | 7 days | 1.79 (0.50 to 6.39) | 1.68 (0.45 to 6.34) |
|  |  |  |  |
|  |  |  |  |
| **Measurement of platelet count** | **Index period** | **Crude** | **Adjusted for medication for  hypertension in history (Yes/No)** |
| **Percentage of hours platelet count ≤20 x109/L** | |  |  |
|  | 1 day | 1.01 (0.21 to 4.88) | 2.21 (0.26 to 18.49) |
|  | 3 days | 0.85 (0.16 to 4.47) | 0.63 (0.10 to 4.02) |
|  | 5 days | 1.90 (0.34 to 10.79) | 1.66 (0.26 to 10.38) |
|  | 7 days | 1.86 (0.30 to 11.56) | 1.97 (0.28 to 14.17) |
| **One or more platelet counts ≤20 x10^9^/L** | |  |  |
|  | 1 day | 3.64 (0.91 to 14.58) | 3.66 (0.85 to 15.78) |
|  | 3 days | 2.33 (0.63 to 8.62) | 2.98 (0.68 to 13.01) |
|  | 5 days | 5.47 (1.08 to 27.75) | 5.86 (0.99 to 34.52) |
|  | 7 days | 4.21 (0.83 to 21.26) | 4.45 (0.79 to 25.15) |
| **One or more platelet counts ≤10 x109/L** | |  |  |
|  | 1 day | 0.67 (0.06 to 7.00) | 0.22 (0.01 to 4.53) |
|  | 3 days | 1.94 (0.44 to 8.56) | 1.68 (0.31 to 8.99) |
|  | 5 days | 1.66 (0.41 to 6.79) | 1.48 (0.29 to 7.52) |
|  | 7 days | 1.79 (0.50 to 6.39) | 1.90 (0.43 to 8.35) |
| **Measurement of platelet count** | **Index period** | **Crude** | **Adjusted for alcohol consumption**  **(Yes/No)** |
| **Percentage of hours platelet count ≤20 x109/L** | |  |  |
|  | 1 day | 1.01 (0.21 to 4.88) | 1.09 (0.19 to 5.97) |
|  | 3 days | 0.85 (0.16 to 4.47) | 0.80 (0.14 to 4.44) |
|  | 5 days | 1.90 (0.34 to 10.79) | 1.87 (0.30 to 11.51) |
|  | 7 days | 1.86 (0.30 to 11.56) | 1.81 (0.27 to 12.14) |
| **One or more platelet counts ≤20 x10^9^/L** | |  |  |
|  | 1 day | 3.64 (0.91 to 14.58) | 3.78 (0.87 to 16.40) |
|  | 3 days | 2.33 (0.63 to 8.62) | 2.54 (0.60 to 10.81) |
|  | 5 days | 5.47 (1.08 to 27.75) | 5.52 (1.04 to 29.48) |
|  | 7 days | 4.21 (0.83 to 21.26) | 4.46 (0.82 to 24.32) |
| **One or more platelet counts ≤10 x109/L** | |  |  |
|  | 1 day | 0.67 (0.06 to 7.00) | 0.70 (0.06 to 8.13) |
|  | 3 days | 1.94 (0.44 to 8.56) | 1.76 (0.38 to 8.12) |
|  | 5 days | 1.66 (0.41 to 6.79) | 1.41 (0.34 to 5.96) |
|  | 7 days | 1.79 (0.50 to 6.39) | 1.59 (0.43 to 5.80) |
| **Measurement of platelet count** | **Index period** | **Crude** | **Adjusted for smoking in history**  **(Ever versus never)** |
| **Percentage of hours platelet count ≤20 x109/L** | |  |  |
|  | 1 day | 1.01 (0.21 to 4.88) | 1.13 (0.22 to 5.90) |
|  | 3 days | 0.85 (0.16 to 4.47) | 0.99 (0.19 to 5.22) |
|  | 5 days | 1.90 (0.34 to 10.79) | 2.11 (0.37 to 12.17) |
|  | 7 days | 1.86 (0.30 to 11.56) | 2.23 (0.34 to 14.68) |
| **One or more platelet counts ≤20 x10^9^/L** | |  |  |
|  | 1 day | 3.64 (0.91 to 14.58) | 3.63 (0.84 to 15.69) |
|  | 3 days | 2.33 (0.63 to 8.62) | 2.70 (0.68 to 10.67) |
|  | 5 days | 5.47 (1.08 to 27.75) | 5.38 (1.05 to 27.42) |
|  | 7 days | 4.21 (0.83 to 21.26) | 3.90 (0.79 to 19.28) |
| **One or more platelet counts ≤10 x109/L** | |  |  |
|  | 1 day | 0.67 (0.06 to 7.00) | 1.20 (0.11 to 13.35) |
|  | 3 days | 1.94 (0.44 to 8.56) | 2.22 (0.50 to 9.92) |
|  | 5 days | 1.66 (0.41 to 6.79) | 1.46 (0.37 to 5.81) |
|  | 7 days | 1.79 (0.50 to 6.39) | 1.66 (0.46 to 5.95) |
| **Measurement of platelet count** | **Index period** | **Crude** | **Adjusted for anti-cholesterol medication usage (ever versus never)** |
| **Percentage of hours platelet count ≤20 x109/L** | |  |  |
|  | 1 day | 1.01 (0.21 to 4.88) | 1.03 (0.19 to 5.51) |
|  | 3 days | 0.85 (0.16 to 4.47) | 0.89 (0.17 to 4.80) |
|  | 5 days | 1.90 (0.34 to 10.79) | 2.04 (0.35 to 11.90) |
|  | 7 days | 1.86 (0.30 to 11.56) | 1.94 (0.31 to 12.38) |
| **One or more platelet counts ≤20 x10^9^/L** | |  |  |
|  | 1 day | 3.64 (0.91 to 14.58) | 4.17 (0.97 to 17.91) |
|  | 3 days | 2.33 (0.63 to 8.62) | 2.72 (0.66 to 11.27) |
|  | 5 days | 5.47 (1.08 to 27.75) | 6.56 (1.16 to 37.14) |
|  | 7 days | 4.21 (0.83 to 21.26) | 5.03 (0.89 to 28.36) |
| **One or more platelet counts ≤10 x109/L** | |  |  |
|  | 1 day | 0.67 (0.06 to 7.00) | 0.69 (0.07 to 7.33) |
|  | 3 days | 1.94 (0.44 to 8.56) | 2.08 (0.46 to 9.47) |
|  | 5 days | 1.66 (0.41 to 6.79) | 1.69 (0.41 to 6.90) |
|  | 7 days | 1.79 (0.50 to 6.39) | 1.84 (0.51 to 6.64) |
|  |  |  |  |
|  |  |  |  |
| **Measurement of platelet count** | **Index period** | **Crude** | **Adjusted for medication for diabetes mellitus in history (ever versus never)** |
| **Percentage of hours platelet count ≤20 x109/L** | |  |  |
|  | 1 day | 1.01 (0.21 to 4.88) | 1.01 (0.21 to 4.88) |
|  | 3 days | 0.85 (0.16 to 4.47) | 0.93 (0.17 to 5.00) |
|  | 5 days | 1.90 (0.34 to 10.79) | 2.07 (0.35 to 12.35) |
|  | 7 days | 1.86 (0.30 to 11.56) | 2.19 (0.33 to 15.55) |
| **One or more platelet counts ≤20 x10^9^/L** | |  |  |
|  | 1 day | 3.64 (0.91 to 14.58) | 3.64 (0.91 to 14.58) |
|  | 3 days | 2.33 (0.63 to 8.62) | 3.07 (0.73 to 12.80) |
|  | 5 days | 5.47 (1.08 to 27.75) | 6.50 (1.19 to 35.57) |
|  | 7 days | 4.21 (0.83 to 21.26) | 5.23 (0.97 to 28.26) |
| **One or more platelet counts ≤10 x109/L** | |  |  |
|  | 1 day | 0.67 (0.06 to 7.00) | 0.67 (0.06 to 7.00) |
|  | 3 days | 1.94 (0.44 to 8.56) | 1.94 (0.44 to 8.56) |
|  | 5 days | 1.66 (0.41 to 6.79) | 1.79 (0.42 to 7.61) |
|  | 7 days | 1.79 (0.50 to 6.39) | 2.17 (0.56 to 8.41) |
| **Measurement of platelet count** | **Index period** | **Crude** | **Adjusted for medication for ischemic heart disease in history (Yes/No)** |
| **Percentage of hours platelet count ≤20 x109/L** | |  |  |
|  | 1 day | 1.01 (0.21 to 4.88) | 1.03 (0.19 to 5.51) |
|  | 3 days | 0.85 (0.16 to 4.47) | 0.91 (0.17 to 4.80) |
|  | 5 days | 1.90 (0.34 to 10.79) | 2.03 (0.35 to 11.70) |
|  | 7 days | 1.86 (0.30 to 11.56) | 1.91 (0.30 to 12.14) |
| **One or more platelet counts ≤20 x10^9^/L** | |  |  |
|  | 1 day | 3.64 (0.91 to 14.58) | 4.13 (0.97 to 17.56) |
|  | 3 days | 2.33 (0.63 to 8.62) | 2.74 (0.67 to 11.30) |
|  | 5 days | 5.47 (1.08 to 27.75) | 6.40 (1.14 to 36.07) |
|  | 7 days | 4.21 (0.83 to 21.26) | 4.89 (0.88 to 27.33) |
| **One or more platelet counts ≤10 x109/L** | |  |  |
|  | 1 day | 0.67 (0.06 to 7.00) | 0.70 (0.07 to 7.37) |
|  | 3 days | 1.94 (0.44 to 8.56) | 2.07 (0.46 to 9.20) |
|  | 5 days | 1.66 (0.41 to 6.79) | 1.64 (0.40 to 6.76) |
|  | 7 days | 1.79 (0.50 to 6.39) | 1.81 (0.50 to 6.53) |
| **Measurement of platelet count** | **Index period** | **Crude** | **Adjusted for graft versus host disease in index period (Yes/No)** |
| **Percentage of hours platelet count ≤20 x109/L** | |  |  |
|  | 1 day | 1.01 (0.21 to 4.88) | 0.79 (0.15 to 4.16) |
|  | 3 days | 0.85 (0.16 to 4.47) | 0.95 (0.18 to 5.10) |
|  | 5 days | 1.90 (0.34 to 10.79) | 2.16 (0.38 to 12.25) |
|  | 7 days | 1.86 (0.30 to 11.56) | 2.12 (0.25 to 13.28) |
| **One or more platelet counts ≤20 x10^9^/L** | |  |  |
|  | 1 day | 3.64 (0.91 to 14.58) | 3.56 (0.14 to 58.59) |
|  | 3 days | 2.33 (0.63 to 8.62) | 2.47 (0.66 to 9.31) |
|  | 5 days | 5.47 (1.08 to 27.75) | 6.04 (1.16 to 31.49) |
|  | 7 days | 4.21 (0.83 to 21.26) | 4.67 (0.90 to 24.32) |
| **One or more platelet counts ≤10 x109/L** | |  |  |
|  | 1 day | 0.67 (0.06 to 7.00) | 0.67 (0.06 to 7.00) |
|  | 3 days | 1.94 (0.44 to 8.56) | 1.85 (0.40 to 8.54) |
|  | 5 days | 1.66 (0.41 to 6.79) | 1.57 (0.37 to 6.71) |
|  | 7 days | 1.79 (0.50 to 6.39) | 1.72 (0.47 to 6.34) |
| **Measurement of platelet count** | **Index period** | **Crude** | **Adjusted for fever ≥38 in index period**  **(per additional day)** |
| **Percentage of hours platelet count ≤20 x109/L** | |  |  |
|  | 1 day | 1.01 (0.21 to 4.88) | 1.10 (0.18 to 6.69) |
|  | 3 days | 0.85 (0.16 to 4.47) | 0.85 (0.16 to 4.56) |
|  | 5 days | 1.90 (0.34 to 10.79) | 1.83 (0.32 to 10.57) |
|  | 7 days | 1.86 (0.30 to 11.56) | 1.80 (0.28 to 11.43) |
| **One or more platelet counts ≤20 x10^9^/L** | |  |  |
|  | 1 day | 3.64 (0.91 to 14.58) | 4.61 (1.06 to 19.98) |
|  | 3 days | 2.33 (0.63 to 8.62) | 2.50 (0.69 to 9.09) |
|  | 5 days | 5.47 (1.08 to 27.75) | 5.31 (1.06 to 26.60) |
|  | 7 days | 4.21 (0.83 to 21.26) | 4.21 (0.84 to 21.06) |
| **One or more platelet counts ≤10 x109/L** | |  |  |
|  | 1 day | 0.67 (0.06 to 7.00) | 0.68 (0.05 to 7.34) |
|  | 3 days | 1.94 (0.44 to 8.56) | 2.34 (0.48 to 11.33) |
|  | 5 days | 1.66 (0.41 to 6.79) | 1.56 (0.36 to 6.88) |
|  | 7 days | 1.79 (0.50 to 6.39) | 1.74 (0.48 to 6.35) |
|  |  |  |  |
|  |  |  |  |
| **Measurement of platelet count** | **Index period** | **Crude** | **Adjusted for fever ≥39 in index period (Per additional day)** |
| **Percentage of hours platelet count ≤20 x109/L** | |  |  |
|  | 1 day | 1.01 (0.21 to 4.88) | NA* |
|  | 3 days | 0.85 (0.16 to 4.47) | 0.81 (0.15 to 4.34) |
|  | 5 days | 1.90 (0.34 to 10.79) | 1.88 (0.33 to 10.61) |
|  | 7 days | 1.86 (0.30 to 11.56) | 1.88 (0.30 to 11.86) |
| **One or more platelet counts ≤20 x10^9^/L** | |  |  |
|  | 1 day | 3.64 (0.91 to 14.58) | NA* |
|  | 3 days | 2.33 (0.63 to 8.62) | 2.35 (0.64 to 8.63) |
|  | 5 days | 5.47 (1.08 to 27.75) | 5.51 (1.09 to 27.92) |
|  | 7 days | 4.21 (0.83 to 21.26) | 4.23 (0.84 to 21.38) |
| **One or more platelet counts ≤10 x109/L** | |  |  |
|  | 1 day | 0.67 (0.06 to 7.00) | NA* |
|  | 3 days | 1.94 (0.44 to 8.56) | 2.00 (0.44 to 9.03) |
|  | 5 days | 1.66 (0.41 to 6.79) | 1.81 (0.05 to 64.88) |
|  | 7 days | 1.79 (0.50 to 6.39) | 1.80 (0.50 to 6.50) |
| **Measurement of platelet count** | **Index period** | **Crude** | **Adjusted for fever ≥38 in index**  **period (Yes/ No)** |
| **Percentage of hours platelet count ≤20 x109/L** | |  |  |
|  | 1 day | 1.01 (0.21 to 4.88) | 1.10 (0.18 to 6.69) |
|  | 3 days | 0.85 (0.16 to 4.47) | 0.95 (0.17 to 5.20) |
|  | 5 days | 1.90 (0.34 to 10.79) | 1.92 (0.33 to 11.08) |
|  | 7 days | 1.86 (0.30 to 11.56) | 1.82 (0.29 to 11.48) |
| **One or more platelet counts ≤20 x10^9^/L** | |  |  |
|  | 1 day | 3.64 (0.91 to 14.58) | 4.61 (1.06 to 19.98) |
|  | 3 days | 2.33 (0.63 to 8.62) | 2.62 (0.71 to 9.72) |
|  | 5 days | 5.47 (1.08 to 27.75) | 5.21 (1.04 to 26.17) |
|  | 7 days | 4.21 (0.83 to 21.26) | 4.21 (0.85 to 20.95) |
| **One or more platelet counts ≤10 x109/L** | |  |  |
|  | 1 day | 0.67 (0.06 to 7.00) | 0.68 (0.06 to 7.34) |
|  | 3 days | 1.94 (0.44 to 8.56) | 2.58 (0.13 to 51.63) |
|  | 5 days | 1.66 (0.41 to 6.79) | 1.68 (0.39 to 7.20) |
|  | 7 days | 1.79 (0.50 to 6.39) | 1.85 (0.51 to 6.69) |
| **Measurement of platelet count** | **Index period** | **Crude** | **Adjusted for fever ≥39 in index  period (Yes/No)** |
| **Percentage of hours platelet count ≤20 x109/L** | |  |  |
|  | 1 day | 1.01 (0.21 to 4.88) | NA* |
|  | 3 days | 0.85 (0.16 to 4.47) | 0.85 (0.16 to 4.45) |
|  | 5 days | 1.90 (0.34 to 10.79) | 1.95 (0.34 (11.26) |
|  | 7 days | 1.86 (0.30 to 11.56) | 1.86 (0.30 to 11.51) |
| **One or more platelet counts ≤20 x10^9^/L** | |  |  |
|  | 1 day | 3.64 (0.91 to 14.58) | NA* |
|  | 3 days | 2.33 (0.63 to 8.62) | 2.34 (0.64 to 8.66) |
|  | 5 days | 5.47 (1.08 to 27.75) | 5.50 (1.08 to 28.17) |
|  | 7 days | 4.21 (0.83 to 21.26) | 4.21 (0.83 to 21.45) |
| **One or more platelet counts ≤10 x109/L** | |  |  |
|  | 1 day | 1.01 (0.21 to 4.88) | NA* |
|  | 3 days | 1.94 (0.44 to 8.56) | 2.02 (0.45 to 9.18) |
|  | 5 days | 1.66 (0.41 to 6.79) | 1.63 (0.40 to 6.72) |
|  | 7 days | 1.79 (0.50 to 6.39) | 1.75 (0.49 to 6.29) |
| **Measurement of platelet count** | **Index period** | **Crude** | **Adjusted for positive culture(s)**  **And/or PCR(s) (Yes/No)** |
| **Percentage of hours platelet count ≤20 x109/L** | |  |  |
|  | 1 day | 1.01 (0.21 to 4.88) | NA* |
|  | 3 days | 0.85 (0.16 to 4.47) | 0.88 (0.16 to 4.70) |
|  | 5 days | 1.90 (0.34 to 10.79) | 2.14 (0.36 to 12.83) |
|  | 7 days | 1.86 (0.30 to 11.56) | 1.85 (0.29 to 11.83) |
| **One or more platelet counts ≤20 x10^9^/L** | |  |  |
|  | 1 day | 3.64 (0.91 to 14.58) | NA* |
|  | 3 days | 2.33 (0.63 to 8.62) | 2.20 (0.61 to 8.03) |
|  | 5 days | 5.47 (1.08 to 27.75) | 4.76 (0.96 to 23.73) |
|  | 7 days | 4.21 (0.83 to 21.26) | 3.73 (0.72 to 19.36) |
| **One or more platelet counts ≤10 x109/L** | |  |  |
|  | 1 day | 0.67 (0.06 to 7.00) | NA* |
|  | 3 days | 1.94 (0.44 to 8.56) | 1.74 (0.38 to 8.15) |
|  | 5 days | 1.66 (0.41 to 6.79) | 1.66 (0.38 to 7.36) |
|  | 7 days | 1.79 (0.50 to 6.39) | 1.56 (0.42 to 5.78) |
|  |  |  |  |
|  |  |  |  |
| **Measurement of platelet count** | **Index period** | **Crude** | **Adjusted for infection treatment**  **in index period (Yes/No)** |
| **Percentage of hours platelet count ≤20 x109/L** | |  |  |
|  | 1 day | 1.01 (0.21 to 4.88) | 1.20 (0.22 to 6.63) |
|  | 3 days | 0.85 (0.16 to 4.47) | 0.85 (0.15 to 4.65) |
|  | 5 days | 1.90 (0.34 to 10.79) | 1.53 (0.23 to 10.01) |
|  | 7 days | 1.86 (0.30 to 11.56) | 1.37 (0.20 to 9.30) |
| **One or more platelet counts ≤20 x10^9^/L** | |  |  |
|  | 1 day | 3.64 (0.91 to 14.58) | 4.12 (0.98 to 17.38) |
|  | 3 days | 2.33 (0.63 to 8.62) | 2.32 (0.61 to 8.80) |
|  | 5 days | 5.47 (1.08 to 27.75) | 4.78 (0.89 to 25.71) |
|  | 7 days | 4.21 (0.83 to 21.26) | 3.87 (0.69 to 21.72) |
| **One or more platelet counts ≤10 x109/L** | |  |  |
|  | 1 day | 0.67 (0.06 to 7.00) | 0.68 (0.07 to 7.04) |
|  | 3 days | 1.94 (0.44 to 8.56) | 1.80 (0.39 to 8.44) |
|  | 5 days | 1.66 (0.41 to 6.79) | 1.30 (0.28 to 6.13) |
|  | 7 days | 1.79 (0.50 to 6.39) | 1.27 (0.32 to 5.09) |
| **Measurement of platelet count** | **Index period** | **Crude** | **Adjusted for maximum leukocyte counts**  **(categories ≤10, 10-25, >25)** |
| **Percentage of hours platelet count ≤20 x109/L** | |  |  |
|  | 1 day | 1.01 (0.21 to 4.88) | NA* |
|  | 3 days | 0.85 (0.16 to 4.47 | 0.78 (0.15 to 4.13) |
|  | 5 days | 1.90 (0.34 to 10.79) | 2.04 (0.35 to 11.85) |
|  | 7 days | 1.86 (0.30 to 11.56) | 1.96 (0.31 to 12.51) |
| **One or more platelet counts ≤20 x10^9^/L** | |  |  |
|  | 1 day | 3.64 (0.91 to 14.58) | NA* |
|  | 3 days | 2.33 (0.63 to 8.62) | 2.11 (0.56 to 7.94) |
|  | 5 days | 5.47 (1.08 to 27.75) | 6.27 (1.04 to 37.89) |
|  | 7 days | 4.21 (0.83 to 21.26) | 4.52 (0.83 to 24.75) |
| **One or more platelet counts ≤10 x109/L** | |  |  |
|  | 1 day | 0.67 (0.06 to 7.00) | NA* |
|  | 3 days | 1.94 (0.44 to 8.56) | 1.94 (0.44 to 8.56) |
|  | 5 days | 1.66 (0.41 to 6.79) | 1.45 (0.35 to 5.96) |
|  | 7 days | 1.79 (0.50 to 6.39) | 1.94 (0.51 to 7.43) |
| **Measurement of platelet count** | **Index period** | **Crude** | **Adjusted for non-intracranial bleeding events in index period (Yes/No)** |
| **Percentage of hours platelet count ≤20 x109/L** | |  |  |
|  | 1 day | 1.01 (0.21 to 4.88) | 0.61 (0.09 to 4.29) |
|  | 3 days | 0.85 (0.16 to 4.47) | 0.34 (0.05 to 2.48) |
|  | 5 days | 1.90 (0.34 to 10.79) | 1.16 (0.17 to 8.03) |
|  | 7 days | 1.86 (0.30 to 11.56) | 1.31 (0.19 to 9.26) |
| **One or more platelet counts ≤20 x10^9^/L** | |  |  |
|  | 1 day | 3.64 (0.91 to 14.58) | 2.78 (0.66 to 11.63) |
|  | 3 days | 2.33 (0.63 to 8.62) | 1.69 (0.41 to 7.00) |
|  | 5 days | 5.47 (1.08 to 27.75) | 4.81 (0.85 to 27.28) |
|  | 7 days | 4.21 (0.83 to 21.26) | 2.75 (0.52 to 14.50) |
| **One or more platelet counts ≤10 x109/L** | |  |  |
|  | 1 day | 0.67 (0.06 to 7.00) | 0.53 (0.05 to 5.43) |
|  | 3 days | 1.94 (0.44 to 8.56) | 1.39 (0.29 to 6.76) |
|  | 5 days | 1.66 (0.41 to 6.79) | 1.26 (0.28 to 5.58) |
|  | 7 days | 1.79 (0.50 to 6.39) | 1.43 (0.36 to 5.61) |
| **Measurement of platelet count** | **Index period** | **Crude** | **Adjusted for trauma (Yes/No; e.g. fall, interventions like lumbar puncture or line placement)** |
| **Percentage of hours platelet count ≤20 x109/L** | |  |  |
|  | 1 day | 1.01 (0.21 to 4.88) | 1.08 (0.21 to 5.54) |
|  | 3 days | 0.85 (0.16 to 4.47) | 1.68 (0.27 to 10.37) |
|  | 5 days | 1.90 (0.34 to 10.79) | 2.65 (0.45 to 15.73) |
|  | 7 days | 1.86 (0.30 to 11.56) | 1.99 (0.30 to 13.28) |
| **One or more platelet counts ≤20 x10^9^/L** | |  |  |
|  | 1 day | 3.64 (0.91 to 14.58) | 4.67 (0.97 to 22.53) |
|  | 3 days | 2.33 (0.63 to 8.62) | 5.04 (0.99 to 25.74) |
|  | 5 days | 5.47 (1.08 to 27.75) | 6.72 (1.24 to 36.33) |
|  | 7 days | 4.21 (0.83 to 21.26) | 3.94 (0.76 to 20.50) |
| **One or more platelet counts ≤10 x109/L** | |  |  |
|  | 1 day | 0.67 (0.06 to 7.00) | 0.75 (0.07 to 8.26) |
|  | 3 days | 1.94 (0.44 to 8.56) | 5.44 (0.59 to 49.70) |
|  | 5 days | 1.66 (0.41 to 6.79) | 2.85 (0.54 to 14.93) |
|  | 7 days | 1.79 (0.50 to 6.39) | 2.47 (0.59 to 10.35) |
|  |  |  |  |
|  |  |  |  |
| **Measurement of platelet count** | **Index period** | **Crude** | **Adjusted for lumbar puncture**  **(*Yes or no)*** |
| **Percentage of hours platelet count ≤20 x109/L** | |  |  |
|  | 1 day | 1.01 (0.21 to 4.88) | NA* |
|  | 3 days | 0.85 (0.16 to 4.47) | NA* |
|  | 5 days | 1.90 (0.34 to 10.79) | 1.91 (0.34 to 10.80) |
|  | 7 days | 1.86 (0.30 to 11.56) | 2.24 (0.33 to 15.02) |
| **One or more platelet counts ≤20 x10^9^/L** | |  |  |
|  | 1 day | 3.64 (0.91 to 14.58) | NA* |
|  | 3 days | 2.33 (0.63 to 8.62) | NA* |
|  | 5 days | 5.47 (1.08 to 27.75) | 5.47 (1.08 to 27.75) |
|  | 7 days | 4.21 (0.83 to 21.26) | 3.98 (0.78 to 20.30) |
| **One or more platelet counts ≤10 x109/L** | |  |  |
|  | 1 day | 0.67 (0.06 to 7.00) | NA* |
|  | 3 days | 1.94 (0.44 to 8.56) | NA* |
|  | 5 days | 1.66 (0.41 to 6.79) | 1.85 (0.43 to 7.97) |
|  | 7 days | 1.79 (0.50 to 6.39) | 2.33 (0.59 to 9.26) |
| **Measurement of platelet count** | **Index period** | **Crude** | **Adjusted for fall (Yes/No)** |
| **Percentage of hours platelet count ≤20 x109/L** | |  |  |
|  | 1 day | 1.01 (0.21 to 4.88) | 1.11 (0.22 to 5.75) |
|  | 3 days | 0.85 (0.16 to 4.47) | 1.08 (0.19 to 6.14) |
|  | 5 days | 1.90 (0.34 to 10.79) | 2.62 (0.43 to 16.03) |
|  | 7 days | 1.86 (0.30 to 11.56) | 2.09 (0.32 (13.46) |
| **One or more platelet counts ≤20 x10^9^/L** | |  |  |
|  | 1 day | 3.64 (0.91 to 14.58) | 5.39 (1.07 to 27.30) |
|  | 3 days | 2.33 (0.63 to 8.62) | 3.41 (0.80 to 14.47) |
|  | 5 days | 5.47 (1.08 to 27.75) | 6.52 (1.24 to 34.33) |
|  | 7 days | 4.21 (0.83 to 21.26) | 4.36 (0.87 to 21.87) |
| **One or more platelet counts ≤10 x109/L** | |  |  |
|  | 1 day | 0.67 (0.06 to 7.00) | 0.73 (0.07 to 7.91) |
|  | 3 days | 1.94 (0.44 to 8.56) | 2.70 (0.49 to 14.83) |
|  | 5 days | 1.66 (0.41 to 6.79) | 1.83 (0.44 to 7.72) |
|  | 7 days | 1.79 (0.50 to 6.39) | 1.79 (0.50 to 6.41) |
| **Measurement of platelet count** | **Index period** | **Crude** | **Adjusted for vomiting in index period (Yes/No)** |
| **Percentage of hours platelet count ≤20 x109/L** | |  |  |
|  | 1 day | 1.01 (0.21 to 4.88) | 0.95 (0.18 to 4.98) |
|  | 3 days | 0.85 (0.16 to 4.47) | 0.53 (0.07 to 3.99) |
|  | 5 days | 1.90 (0.34 to 10.79) | 1.15 (0.16 to 8.07) |
|  | 7 days | 1.86 (0.30 to 11.56) | 1.34 (0.20 to 9.08) |
| **One or more platelet counts ≤20 x10^9^/L** | |  |  |
|  | 1 day | 3.64 (0.91 to 14.58) | 3.58 (0.87 to 14.77) |
|  | 3 days | 2.33 (0.63 to 8.62) | 2.17 (0.54 to 8.73) |
|  | 5 days | 5.47 (1.08 to 27.75) | 4.44 (0.81 to 24.50) |
|  | 7 days | 4.21 (0.83 to 21.26) | 3.58 (0.66 to 19.33) |
| **One or more platelet counts ≤10 x109/L** | |  |  |
|  | 1 day | 0.67 (0.06 to 7.00) | 0.42 (0.03 to 6.62) |
|  | 3 days | 1.94 (0.44 to 8.56) | 1.72 (0.36 to 8.22) |
|  | 5 days | 1.66 (0.41 to 6.79) | 1.57 (0.35 to 6.99) |
|  | 7 days | 1.79 (0.50 to 6.39) | 1.76 (0.48 to 6.52) |
| **Measurement of platelet count** | **Index period** | **Crude** | **Adjusted for number of RBC**  **transfusions** |
| **Percentage of hours platelet count ≤20 x109/L** | |  |  |
|  | 1 day | 1.01 (0.21 to 4.88) | 1.03 (0.21 to 5.17) |
|  | 3 days | 0.85 (0.16 to 4.47) | 0.81 (0.14 to 4.70) |
|  | 5 days | 1.66 (0.41 to 6.79) | 2.14 (0.33 to 13.63) |
|  | 7 days | 1.86 (0.30 to 11.56) | 2.03 (0.30 to 13.87) |
| **One or more platelet counts ≤20 x10^9^/L** | |  |  |
|  | 1 day | 3.64 (0.91 to 14.58) | 4.24 (0.95 to 18.88) |
|  | 3 days | 2.33 (0.63 to 8.62) | 2.21 (0.58 to 8.43) |
|  | 5 days | 5.47 (1.08 to 27.75) | 4.89 (0.93 to 25.73) |
|  | 7 days | 4.21 (0.83 to 21.26) | 3.60 (0.69 to 18.90) |
| **One or more platelet counts ≤10 x109/L** | |  |  |
|  | 1 day | 0.67 (0.06 to 7.00) | 0.67 (0.06 to 6.99) |
|  | 3 days | 1.94 (0.44 to 8.56) | 1.86 (0.41 to 8.49) |
|  | 5 days | 1.66 (0.41 to 6.79) | 1.66 (0.37 to 7.37) |
|  | 7 days | 1.79 (0.50 to 6.39) | 1.77 (0.47 to 6.59) |
|  |  |  |  |
|  |  |  |  |
| **Measurement of platelet count** | **Index period** | **Crude** | **Adjusted for**  **RBC transfusions categorical** |
| **Percentage of hours platelet count ≤20 x109/L** | |  |  |
|  | 1 day | 1.01 (0.21 to 4.88) | 0.98 (0.20 to 4.73) |
|  | 3 days | 0.85 (0.16 to 4.47 | 0.93 (0.16 to 5.60) |
|  | 5 days | 1.90 (0.34 to 10.79) | 2.03 (0.32 to 12.69) |
|  | 7 days | 1.86 (0.30 to 11.56) | 1.77 (0.27 to 11.86) |
| **One or more platelet counts ≤20 x10^9^/L** | |  |  |
|  | 1 day | 3.64 (0.91 to 14.58) | 3.86 (0.88 to 17.08) |
|  | 3 days | 2.33 (0.63 to 8.62) | 2.10 (0.53 to 8.24) |
|  | 5 days | 5.47 (1.08 to 27.75) | 7.42 (1.15 to 47.78) |
|  | 7 days | 4.21 (0.83 to 21.26) | 4.81 (0.80 to 28.81) |
| **One or more platelet counts ≤10 x109/L** | |  |  |
|  | 1 day | 0.67 (0.06 to 7.00) | 0.68 (0.07 to 7.00) |
|  | 3 days | 1.94 (0.44 to 8.56) | 1.92 (0.43 to 8.61) |
|  | 5 days | 1.66 (0.41 to 6.79) | 1.53 (0.36 to 6.53) |
|  | 7 days | 1.79 (0.50 to 6.39) | 1.69 (0.45 to 6.26) |

*NA: conditional logistic regression model did not run due to no disconcordant groups or too little events

# Table S4. Crude and adjusted rate ratios for the association between platelet transfusions and the incidence of intracranial hemorrhage among patients with acute leukemia

|  |  | **RR (95% CI)** | | | | | | |
| --- | --- | --- | --- | --- | --- | --- | --- | --- |
|  | **Implicated time period** | **Category** | **n** | **Crude** | **adjusted for one or more platelet count ≤10 x10^9^/L** | **adjusted for one or more platelet count ≤20 x10^9^/L** | **adjusted for % of hours platelet count ≤20 x10^9^/L** |  |
| **Platelet transfusions** | | | | | | | |  |
|  | **1 day** | **0** | 52 | Ref | Ref | Ref | Ref |  |
|  |  | **1 to 2** | 20 | 3.86 (1.08 to 13.79) | 4.50 (1.20 to 16.90) | 2.67 (0.63 to 11.33) | 2.32 (0.59 to 9.17) |  |
|  |  | **>2** | 0 | - | - |  | - |  |
|  | **3 days** | **0** | 39 | Ref | Ref | Ref | Ref |  |
|  |  | **1 to 2** | 23 | 2.32 (0.60 to 9.01) | 2.36 (0.54 to 10.40) | 2.04 (0.43 to 9.59) | 2.94 (0.69 to 12.55) |  |
|  |  | **>2** | 10 | 8.12 (0.80 to 82.2) | 8.27 (0.73 to 93.51) | 6.96 (0.59 to 82.31) | 9.74 (0.92 to 103.04) |  |
|  | **5 days** | **0** | 30 | Ref | Ref | Ref | Ref |  |
|  |  | **1 to 2** | 24 | 2.16 (0.37 to 12.55) | 2.21 (0.32 to 15.23) | 1.41 (0.20 to 9.98) | 2.14 (0.35 to 13.08) |  |
|  |  | **>2** | 18 | 13.11 (1.91 to 90.03) | 13.36 (1.78 to 100.28) | 7.45 (0.88 to 63.07) | 12.99 (1.81 to 93.49) |  |
|  | **7 days** | **0** | 23 | Ref | Ref | Ref | Ref |  |
|  |  | **1 to 2** | 26 | 4.04 (0.73 to 22.27) | 4.09 (0.70 to 23.85) | 3.01 (0.44 to 20.48 | 4.06 (0.71 to 23.18) |  |
|  |  | **>2** | 23 | 8.91 (1.53 to 51.73) | 9.02 (1.47 to 55.49) | 6.50 (0.93 to 45.42) | 8.94 (1.49 to 53.69) |  |

# Table S5. Crude and adjusted rate ratios*, for the association between platelet transfusions (on a continuous scale) and the incidence of intracranial hemorrhage among patients with acute leukemia

| **Implicated time period** | **Crude** | **adjusted for one or more platelet counts ≤10 x10^9^/L** | **adjusted for one or more platelet counts ≤20 x10^9^/L** | **adjusted for % of hours PLT ≤20 x10^9^/L** |
| --- | --- | --- | --- | --- |
| **1 day** | 2.46 (1.02 to 5.91) | 2.61 (1.07 to 6.36) | 1.99 (0.78 to 5.07) | 1.80 (0.75 to 4.34) |
| **3 days** | 1.92 (1.10 to 3.36) | 1.91 (1.08 to 3.40) | 1.90 (1.01 to 3.58) | 2.01 (1.18 to 3.62) |
| **5 days** | 1.60 (1.08 to 2.37) | 1.58 (1.06 to 2.36) | 1.46 (0.99 to 2.15) | 1.58 (1.07 to 2.34) |
| **7 days** | 1.48 (1.06 to 2.07) | 1.47 (1.05 to 2.05) | 1.40 (1.01 to 1.95) | 1.47 (1.06 to 2.05) |

*Rate ratios are to be interpreted as continuous, thus for example one platelet transfusion has a crude RR of 1.48^1^ for the seven-day period, two transfusions 1.48^2^, three transfusions 1.48^3^ and so on.

# Table S6. Crude rate ratios (RR) for platelet (PLT) transfusions on ICH and adjusted RR’s for clinical risk factors.

| **Measurement of platelet transfusion** | **Index period, category** | **Crude** | **Adjusted for sex**  **female (male is ref)** |
| --- | --- | --- | --- |
| **Platelet transfusion, continuous** | |  |  |
|  | 1 day | 2.46 (1.02 to 5.91) | 2.43 (0.99 to 6.01) |
|  | 3 days | 1.92 (1.10 to 3.36) | 1.91 (1.09 to 3.37) |
|  | 5 days | 1.60 (1.08 to 2.37) | 1.60 (1.07 to 2.38) |
|  | 7 days | 1.48 (1.06 to 2.07) | 1.49 (1.06 to 2.09) |
| **Platelet transfusion, categorical (ref=0)** | |  |  |
|  | 1 day, 1 to 2 PLT transfusions | 3.86 (1.08 to 13.79) | 3.77 (1.04 to 13.63) |
|  | 1 day, > 2 PLT transfusions | NA* | NA* |
|  | 3 days, 1 to 2 PLT transfusions | 2.32 (0.60 to 9.01) | 2.34 (0.60 to 9.09) |
|  | 3 days, > 2 PLT transfusions | 8.12 (0.80 to 82.2) | 7.68 (0.75 to 78.74) |
|  | 5 days, 1 to 2 PLT transfusions | 2.16 (0.37 to 12.55) | 2.10 (0.36 to 12.23) |
|  | 5 days, > 2 PLT transfusions | 13.11 (1.91 to 90.03) | 13.10 (1.91 to 89.76) |
|  | 7 days, 1 to 2 PLT transfusions | 4.04 (0.73 to 22.27) | 3.85 (0.69 to 21.43) |
|  | 7 days, > 2 PLT transfusions | 8.91 (1.53 to 51.73) | 8.79 (1.50 to 51.36) |
| **Measurement of platelet transfusion** | **Index period** | **Crude** | **Adjusted for age  (per additional year)** |
| **Platelet transfusion, continuous** | |  |  |
|  | 1 day | 2.46 (1.02 to 5.91) | 2.50 (1.01 to 6.19) |
|  | 3 days | 1.92 (1.10 to 3.36) | 1.83 (1.04 to 3.23) |
|  | 5 days | 1.60 (1.08 to 2.37) | 1.56 (1.04 to 2.33) |
|  | 7 days | 1.48 (1.06 to 2.07) | 1.46 (1.04 to 2.05) |
| **Platelet transfusion, categorical (ref=0)** | |  |  |
|  | 1 day, 1 to 2 PLT transfusions | 3.86 (1.08 to 13.79) | 3.95 (1.04 to 14.98) |
|  | 1 day, > 2 PLT transfusions | NA* | NA* |
|  | 3 days, 1 to 2 PLT transfusions | 2.32 (0.60 to 9.01) | 2.15 (0.52 to 8.86) |
|  | 3 days, > 2 PLT transfusions | 8.12 (0.80 to 82.2) | 6.41 (0.61 to 67.71) |
|  | 5 days, 1 to 2 PLT transfusions | 2.16 (0.37 to 12.55) | 2.20 (0.33 to 14.52) |
|  | 5 days, > 2 PLT transfusions | 13.11 (1.91 to 90.03) | 13.30 (1.75 to 101.10) |
|  | 7 days, 1 to 2 PLT transfusions | 4.04 (0.73 to 22.27) | 4.95 (0.77 to 31.77) |
|  | 7 days, > 2 PLT transfusions | 8.91 (1.53 to 51.73) | 11.25 (1.65 to 76.50) |
| **Measurement of platelet transfusion** | **Index period** | **Crude** | **Adjusted for antiplatelet or  therapeutic anticoagulation  medication within 10 days (Yes/No)** |
| **Platelet transfusion, continuous** | |  |  |
|  | 1 day | 2.46 (1.02 to 5.91) | 2.64 (1.05 to 6.66) |
|  | 3 days | 1.92 (1.10 to 3.36) | 1.73 (0.98 to 3.07) |
|  | 5 days | 1.60 (1.08 to 2.37) | 1.44 (0.96 to 2.18) |
|  | 7 days | 1.48 (1.06 to 2.07) | 1.39 (0.98 to 7.97) |
| **Platelet transfusion, categorical (ref=0)** | |  |  |
|  | 1 day, 1 to 2 PLT transfusions | 3.86 (1.08 to 13.79) | 4.31 (1.08 to 17.19) |
|  | 1 day, > 2 PLT transfusions | NA* | NA* |
|  | 3 days, 1 to 2 PLT transfusions | 2.32 (0.60 to 9.01) | 1.93 (0.46 to 8.01) |
|  | 3 days, > 2 PLT transfusions | 8.12 (0.80 to 82.2) | 5.75 (0.57 to 58.09) |
|  | 5 days, 1 to 2 PLT transfusions | 2.16 (0.37 to 12.55) | 2.14 (0.30 to 15.07) |
|  | 5 days, > 2 PLT transfusions | 13.11 (1.91 to 90.03) | 7.99 (0.99 to 64.70) |
|  | 7 days, 1 to 2 PLT transfusions | 4.04 (0.73 to 22.27) | 3.76 (0.60 to 23.56) |
|  | 7 days, > 2 PLT transfusions | 8.91 (1.53 to 51.73) | 7.91 (1.16 to 54.00) |
| **Measurement of platelet transfusion** | **Index period** | **Crude** | **Adjusted for cardiovascular disease  risk score (per additional risk factor)** |
| **Platelet transfusion, continuous** | |  |  |
|  | 1 day | 2.46 (1.02 to 5.91) | 2.58 (1.01 to 6.61) |
|  | 3 days | 1.92 (1.10 to 3.36) | 1.75 (1.01 to 3.05) |
|  | 5 days | 1.60 (1.08 to 2.37) | 1.42 (0.98 to 2.08) |
|  | 7 days | 1.48 (1.06 to 2.07) | 1.36 (0.99 to 7.88) |
| **Platelet transfusion, categorical (ref=0)** | |  |  |
|  | 1 day, 1 to 2 PLT transfusions | 3.86 (1.08 to 13.79) | 4.04 (1.00 to 16.36) |
|  | 1 day, > 2 PLT transfusions | NA* | NA* |
|  | 3 days, 1 to 2 PLT transfusions | 2.32 (0.60 to 9.01) | 2.19 (0.54 to 8.88) |
|  | 3 days, > 2 PLT transfusions | 8.12 (0.80 to 82.2) | 6.27 (0.61 to 64.19) |
|  | 5 days, 1 to 2 PLT transfusions | 2.16 (0.37 to 12.55) | 2.18 (0.35 to 13.51) |
|  | 5 days, > 2 PLT transfusions | 13.11 (1.91 to 90.03) | 9.37 (1.22 to 72.22) |
|  | 7 days, 1 to 2 PLT transfusions | 4.04 (0.73 to 22.27) | 4.44 (0.75 to 26.36) |
|  | 7 days, > 2 PLT transfusions | 8.91 (1.53 to 51.73) | 7.32 (1.18 to 45.36) |
|  |  |  |  |
|  |  |  |  |
| **Measurement of platelet transfusion** | **Index period** | **Crude** | **Adjusted for medication for  hypertension in history (Yes/No)** |
| **Platelet transfusion, continuous** | |  |  |
|  | 1 day | 2.46 (1.02 to 5.91) | 3.05 (0.99 to 9.37) |
|  | 3 days | 1.92 (1.10 to 3.36) | 1.96 (1.02 to 3.76) |
|  | 5 days | 1.60 (1.08 to 2.37) | 1.49 (0.98 to 2.26) |
|  | 7 days | 1.48 (1.06 to 2.07) | 1.42 (0.99 to 2.02) |
| **Platelet transfusion, categorical (ref=0)** | |  |  |
|  | 1 day, 1 to 2 PLT transfusions | 3.86 (1.08 to 13.79) | 4.18 (0.93 to 18.84) |
|  | 1 day, > 2 PLT transfusions | NA* | NA* |
|  | 3 days, 1 to 2 PLT transfusions | 2.32 (0.60 to 9.01) | 2.30 (0.53 to 10.05) |
|  | 3 days, > 2 PLT transfusions | 8.12 (0.80 to 82.2) | 10.38 (0.51 to 213.14) |
|  | 5 days, 1 to 2 PLT transfusions | 2.16 (0.37 to 12.55) | 2.86 (0.36 to 23.01) |
|  | 5 days, > 2 PLT transfusions | 13.11 (1.91 to 90.03) | 14.46 (1.43 to 146.20) |
|  | 7 days, 1 to 2 PLT transfusions | 4.04 (0.73 to 22.27) | 4.23 (0.64 to 28.16) |
|  | 7 days, > 2 PLT transfusions | 8.91 (1.53 to 51.73) | 10.92 (1.45 to 82.44) |
| **Measurement of platelet transfusion** | **Index period** | **Crude** | **Adjusted for alcohol consumption**  **(Yes/No)** |
| **Platelet transfusion, continuous** | |  |  |
|  | 1 day | 2.46 (1.02 to 5.91) | 2.57 (1.02 to 6.51) |
|  | 3 days | 1.92 (1.10 to 3.36) | 1.98 (1.09 to 3.62) |
|  | 5 days | 1.60 (1.08 to 2.37) | 1.63 (1.07 to 2.47) |
|  | 7 days | 1.48 (1.06 to 2.07) | 1.50 (1.06 to 2.11) |
| **Platelet transfusion, categorical (ref=0)** | |  |  |
|  | 1 day, 1 to 2 PLT transfusions | 3.86 (1.08 to 13.79) | 4.39 (1.10 to 17.55) |
|  | 1 day, > 2 PLT transfusions | NA* | NA* |
|  | 3 days, 1 to 2 PLT transfusions | 2.32 (0.60 to 9.01) | 2.59 (0.62 to 10.88) |
|  | 3 days, > 2 PLT transfusions | 8.12 (0.80 to 82.2) | 9.32 (0.75 to 115.47) |
|  | 5 days, 1 to 2 PLT transfusions | 2.16 (0.37 to 12.55) | 2.24 (0.38 to 13.19) |
|  | 5 days, > 2 PLT transfusions | 13.11 (1.91 to 90.03) | 16.07 (2.08 to 124.11) |
|  | 7 days, 1 to 2 PLT transfusions | 4.04 (0.73 to 22.27) | 4.11 (0.73 to 23.17) |
|  | 7 days, > 2 PLT transfusions | 8.91 (1.53 to 51.73) | 9.45 (1.56 to 57.15) |
| **Measurement of platelet transfusion** | **Index period** | **Crude** | **Adjusted for smoking in history**  **(Ever versus never)** |
| **Platelet transfusion, continuous** | |  |  |
|  | 1 day | 2.46 (1.02 to 5.91) | 3.03 (1.12 to 8.20) |
|  | 3 days | 1.92 (1.10 to 3.36) | 2.06 (1.11 to 3.82) |
|  | 5 days | 1.60 (1.08 to 2.37) | 1.60 (1.06 to 2.42) |
|  | 7 days | 1.48 (1.06 to 2.07) | 1.47 (1.05 to 2.04) |
| **Platelet transfusion, categorical (ref=0)** | |  |  |
|  | 1 day, 1 to 2 PLT transfusions | 3.86 (1.08 to 13.79) | 4.95 (1.15 to 21.26) |
|  | 1 day, > 2 PLT transfusions | NA* | NA* |
|  | 3 days, 1 to 2 PLT transfusions | 2.32 (0.60 to 9.01) | 2.12 (0.50 to 8.94) |
|  | 3 days, > 2 PLT transfusions | 8.12 (0.80 to 82.2) | 12.24 (0.97 to 154.98) |
|  | 5 days, 1 to 2 PLT transfusions | 2.16 (0.37 to 12.55) | 1.88 (0.30 to 11.90) |
|  | 5 days, > 2 PLT transfusions | 13.11 (1.91 to 90.03) | 16.25 (1.74 to 151.89) |
|  | 7 days, 1 to 2 PLT transfusions | 4.04 (0.73 to 22.27) | 3.95 (0.68 to 22.92) |
|  | 7 days, > 2 PLT transfusions | 8.91 (1.53 to 51.73) | 8.43 (1.29 to 54.96) |
| **Measurement of platelet transfusion** | **Index period** | **Crude** | **Adjusted for anti to cholesterol medication usage (ever versus never)** |
| **Platelet transfusion, continuous** | |  |  |
|  | 1 day | 2.46 (1.02 to 5.91) | 2.54 (1.05 to 6.14) |
|  | 3 days | 1.92 (1.10 to 3.36) | 1.97 (1.11 to 3.51) |
|  | 5 days | 1.60 (1.08 to 2.37) | 1.60 (1.08 to 2.37) |
|  | 7 days | 1.48 (1.06 to 2.07) | 1.48 (1.06 to 2.07) |
| **Platelet transfusion, categorical (ref=0)** | |  |  |
|  | 1 day, 1 to 2 PLT transfusions | 3.86 (1.08 to 13.79) | 9.99 (1.10 to 14.50) |
|  | 1 day, > 2 PLT transfusions | NA* | NA* |
|  | 3 days, 1 to 2 PLT transfusions | 2.32 (0.60 to 9.01) | 2.53 (0.63 to 10.25) |
|  | 3 days, > 2 PLT transfusions | 8.12 (0.80 to 82.2) | 8.46 (0.83 to 86.30) |
|  | 5 days, 1 to 2 PLT transfusions | 2.16 (0.37 to 12.55) | 2.16 (0.37 to 12.56) |
|  | 5 days, > 2 PLT transfusions | 13.11 (1.91 to 90.03) | 13.11 (1.91 to 90.12) |
|  | 7 days, 1 to 2 PLT transfusions | 4.04 (0.73 to 22.27) | 4.03 (0.73 to 22.31) |
|  | 7 days, > 2 PLT transfusions | 8.91 (1.53 to 51.73) | 8.90 (1.53 to 51.77) |
|  |  |  |  |
|  |  |  |  |
| **Measurement of platelet transfusion** | **Index period** | **Crude** | **Adjusted for medication for diabetes mellitus in history (ever versus never)** |
| **Platelet transfusion, continuous** | |  |  |
|  | 1 day | 2.46 (1.02 to 5.91) | 2.55 (1.03 to 6.29) |
|  | 3 days | 1.92 (1.10 to 3.36) | 2.05 (1.15 to 3.64) |
|  | 5 days | 1.60 (1.08 to 2.37) | 1.64 (1.12 to 2.40) |
|  | 7 days | 1.48 (1.06 to 2.07) | 1.51 (1.10 to 2.07) |
| **Platelet transfusion, categorical (ref=0)** | |  |  |
|  | 1 day, 1 to 2 PLT transfusions | 3.86 (1.08 to 13.79) | 3.86 (1.04 to 14.34) |
|  | 1 day, > 2 PLT transfusions | NA* | NA* |
|  | 3 days, 1 to 2 PLT transfusions | 2.32 (0.60 to 9.01) | 2.39 (0.59 to 9.59) |
|  | 3 days, > 2 PLT transfusions | 8.12 (0.80 to 82.2) | 11.76 (0.90 to 154.32) |
|  | 5 days, 1 to 2 PLT transfusions | 2.16 (0.37 to 12.55) | 2.48 (0.40 to 15.23) |
|  | 5 days, > 2 PLT transfusions | 13.11 (1.91 to 90.03) | 14.84 (2.05 to 107.67) |
|  | 7 days, 1 to 2 PLT transfusions | 4.04 (0.73 to 22.27) | 3.41 (0.61 to 18.89) |
|  | 7 days, > 2 PLT transfusions | 8.91 (1.53 to 51.73) | 8.29 (1.45 to 47.49) |
| **Measurement of platelet transfusion** | **Index period** | **Crude** | **Adjusted for medication for ischemic heart disease in history (Yes/No)** |
| **Platelet transfusion, continuous** | |  |  |
|  | 1 day | 2.46 (1.02 to 5.91) | 2.51 (1.04 to 6.04) |
|  | 3 days | 1.92 (1.10 to 3.36) | 1.97 (1.11 to 3.50) |
|  | 5 days | 1.60 (1.08 to 2.37) | 1.59 (1.07 to 2.36) |
|  | 7 days | 1.48 (1.06 to 2.07) | 1.48 (1.06 to 2.07) |
| **Platelet transfusion, categorical (ref=0)** | |  |  |
|  | 1 day, 1 to 2 PLT transfusions | 3.86 (1.08 to 13.79) | 3.96 (1.09 to 14.38) |
|  | 1 day, > 2 PLT transfusions | NA* | NA* |
|  | 3 days, 1 to 2 PLT transfusions | 2.32 (0.60 to 9.01) | 2.52 (0.63 to 10.12) |
|  | 3 days, > 2 PLT transfusions | 8.12 (0.80 to 82.2) | 8.44 (0.83 to 86.00) |
|  | 5 days, 1 to 2 PLT transfusions | 2.16 (0.37 to 12.55) | 2.16 (0.37 to 12.53) |
|  | 5 days, > 2 PLT transfusions | 13.11 (1.91 to 90.03) | 13.05 (1.89 to 90.05) |
|  | 7 days, 1 to 2 PLT transfusions | 4.04 (0.73 to 22.27) | 4.00 (0.72 to 22.05) |
|  | 7 days, > 2 PLT transfusions | 8.91 (1.53 to 51.73) | 8.81 (1.50 to 51.60) |
| **Measurement of platelet transfusion** | **Index period** | **Crude** | **Adjusted for graft versus host disease in index period (Yes/No)** |
| **Platelet transfusion, continuous** | |  |  |
|  | 1 day | 2.46 (1.02 to 5.91) | 2.45 (1.01 to 5.91) |
|  | 3 days | 1.92 (1.10 to 3.36) | 1.90 (1.09 to 3.30) |
|  | 5 days | 1.60 (1.08 to 2.37) | 1.58 (1.06 to 2.34) |
|  | 7 days | 1.48 (1.06 to 2.07) | 1.47 (1.05 to 2.05) |
| **Platelet transfusion, categorical (ref=0)** | |  |  |
|  | 1 day, 1 to 2 PLT transfusions | 3.86 (1.08 to 13.79) | 3.72 (1.02 to 13.58) |
|  | 1 day, > 2 PLT transfusions | NA* | NA* |
|  | 3 days, 1 to 2 PLT transfusions | 2.32 (0.60 to 9.01) | 1.97 (0.49 to 7.98) |
|  | 3 days, > 2 PLT transfusions | 8.12 (0.80 to 82.2) | 9.07 (0.85 to 96.73) |
|  | 5 days, 1 to 2 PLT transfusions | 2.16 (0.37 to 12.55) | 1.87 (0.31 to 11.27) |
|  | 5 days, > 2 PLT transfusions | 13.11 (1.91 to 90.03) | 12.09 (1.74 to 84.04) |
|  | 7 days, 1 to 2 PLT transfusions | 4.04 (0.73 to 22.27) | 3.82 (0.66 to 22.03) |
|  | 7 days, > 2 PLT transfusions | 8.91 (1.53 to 51.73) | 8.46 (1.40 to 51.23) |
| **Measurement of platelet transfusion** | **Index period** | **Crude** | **Adjusted for fever ≥38 in index period**  **(per additional day)** |
| **Platelet transfusion, continuous** | |  |  |
|  | 1 day | 2.46 (1.02 to 5.91) | 2.96 (1.08 to 8.08) |
|  | 3 days | 1.92 (1.10 to 3.36) | 2.23 (1.18 to 4.21) |
|  | 5 days | 1.60 (1.08 to 2.37) | 1.85 (1.15 to 2.98) |
|  | 7 days | 1.48 (1.06 to 2.07) | 1.64 (1.13 to 2.40) |
| **Platelet transfusion, categorical (ref=0)** | |  |  |
|  | 1 day, 1 to 2 PLT transfusions | 3.86 (1.08 to 13.79) | 6.82 (1.40 to 33.14) |
|  | 1 day, > 2 PLT transfusions | NA* | NA* |
|  | 3 days, 1 to 2 PLT transfusions | 2.32 (0.60 to 9.01) | 3.13 (0.75 to 13.12) |
|  | 3 days, > 2 PLT transfusions | 8.12 (0.80 to 82.2) | 11.66 (1.03 to 131.71) |
|  | 5 days, 1 to 2 PLT transfusions | 2.16 (0.37 to 12.55) | 3.16 (0.43 to 23.05) |
|  | 5 days, > 2 PLT transfusions | 13.11 (1.91 to 90.03) | 48.07 (2.70 to 856.31) |
|  | 7 days, 1 to 2 PLT transfusions | 4.04 (0.73 to 22.27) | 3.75 (0.68 to 20.75) |
|  | 7 days, > 2 PLT transfusions | 8.91 (1.53 to 51.73) | 11.77 (1.77 to 78.10) |
|  |  |  |  |
|  |  |  |  |
| **Measurement of platelet transfusion** | **Index period** | **Crude** | **Adjusted for fever ≥39 in index period (Per additional day)** |
| **Platelet transfusion, continuous** | |  |  |
|  | 1 day | 2.46 (1.02 to 5.91) | NA |
|  | 3 days | 1.92 (1.10 to 3.36) | 2.15 (1.14 to 4.04) |
|  | 5 days | 1.60 (1.08 to 2.37) | 1.69 (1.13 to 2.52) |
|  | 7 days | 1.48 (1.06 to 2.07) | 1.55 (1.11 to 2.17) |
| **Platelet transfusion, categorical (ref=0)** | |  |  |
|  | 1 day, 1 to 2 PLT transfusions | 3.86 (1.08 to 13.79) | NA* |
|  | 1 day, > 2 PLT transfusions | NA* | NA* |
|  | 3 days, 1 to 2 PLT transfusions | 2.32 (0.60 to 9.01) | 2.42 (0.63 to 9.32) |
|  | 3 days, > 2 PLT transfusions | 8.12 (0.80 to 82.2) | 9.26 (0.89 to 96.80) |
|  | 5 days, 1 to 2 PLT transfusions | 2.16 (0.37 to 12.55) | 2.06 (0.36 to 11.89) |
|  | 5 days, > 2 PLT transfusions | 13.11 (1.91 to 90.03) | 28.04 (2.68 to 293.33) |
|  | 7 days, 1 to 2 PLT transfusions | 4.04 (0.73 to 22.27) | 3.89 (0.71 to 71.53) |
|  | 7 days, > 2 PLT transfusions | 8.91 (1.53 to 51.73) | 11.35 (1.80 to 71.53) |
| **Measurement of platelet transfusion** | **Index period** | **Crude** | **Adjusted for fever ≥38 in index**  **period (Yes/ No)** |
| **Platelet transfusion, continuous** | |  |  |
|  | 1 day | 2.46 (1.02 to 5.91) | 2,96 (1.08 to 8.08) |
|  | 3 days | 1.92 (1.10 to 3.36) | 2.07 (1.14 to 3.75) |
|  | 5 days | 1.60 (1.08 to 2.37) | 1.69 (1.11 to 2.56) |
|  | 7 days | 1.48 (1.06 to 2.07) | 1.56 (1.09 to 2.24) |
| **Platelet transfusion, categorical (ref=0)** | |  |  |
|  | 1 day, 1 to 2 PLT transfusions | 3.86 (1.08 to 13.79) | 6.82 (1.40 to 33.14) |
|  | 1 day, > 2 PLT transfusions | NA* | NA* |
|  | 3 days, 1 to 2 PLT transfusions | 2.32 (0.60 to 9.01) | 3.20 (0.73 to 14.01) |
|  | 3 days, > 2 PLT transfusions | 8.12 (0.80 to 82.2) | 12.18 (1.10 to 135.38) |
|  | 5 days, 1 to 2 PLT transfusions | 2.16 (0.37 to 12.55) | 3.60 (0.50 to 26.04) |
|  | 5 days, > 2 PLT transfusions | 13.11 (1.91 to 90.03) | 25.22 (2.38 to 266.93) |
|  | 7 days, 1 to 2 PLT transfusions | 4.04 (0.73 to 22.27) | 4.11 (0.74 to 22.67) |
|  | 7 days, > 2 PLT transfusions | 8.91 (1.53 to 51.73) | 10.68 (1.70 to 67.08) |
| **Measurement of platelet transfusion** | **Index period** | **Crude** | **Adjusted for fever ≥39 in index period (Yes/No)** |
| **Platelet transfusion, continuous** | |  |  |
|  | 1 day | 2.46 (1.02 to 5.91) | NA |
|  | 3 days | 1.92 (1.10 to 3.36) | 2.15 (1.14 to 4.04) |
|  | 5 days | 1.60 (1.08 to 2.37) | 1.65 (1.10 to 2.47) |
|  | 7 days | 1.48 (1.06 to 2.07) | 1.54 (1.09 to 2.17) |
| **Platelet transfusion, categorical (ref=0)** | |  |  |
|  | 1 day, 1 to 2 PLT transfusions | 3.86 (1.08 to 13.79) | NA* |
|  | 1 day, > 2 PLT transfusions | NA* | NA* |
|  | 3 days, 1 to 2 PLT transfusions | 2.32 (0.60 to 9.01) | 2.42 (0.63 to 9.32) |
|  | 3 days, > 2 PLT transfusions | 8.12 (0.80 to 82.2) | 9.26 (0.89 to 96.80) |
|  | 5 days, 1 to 2 PLT transfusions | 2.16 (0.37 to 12.55) | 2.10 (0.36 to 12.07) |
|  | 5 days, > 2 PLT transfusions | 13.11 (1.91 to 90.03) | 19.20 (2.24 to 164.44) |
|  | 7 days, 1 to 2 PLT transfusions | 4.04 (0.73 to 22.27) | 3.89 (0.70 to 21.53) |
|  | 7 days, > 2 PLT transfusions | 8.91 (1.53 to 51.73) | 10.35 (1.62 to 66.24) |
| **Measurement of platelet transfusion** | **Index period** | **Crude** | **Adjusted for positive culture(s)**  **And/or PCR(s) (Yes/No)** |
| **Platelet transfusion, continuous** | |  |  |
|  | 1 day | 2.46 (1.02 to 5.91) | 2.40 (0.99 to 5.82) |
|  | 3 days | 1.92 (1.10 to 3.36) | 1.84 (1.03 to 3.29) |
|  | 5 days | 1.60 (1.08 to 2.37) | 1.52 (1.03 to 2.23) |
|  | 7 days | 1.48 (1.06 to 2.07) | 1.48 (1.04 to 2.11) |
| **Platelet transfusion, categorical (ref=0)** | |  |  |
|  | 1 day, 1 to 2 PLT transfusions | 3.86 (1.08 to 13.79) | 3.74 (1.04 to 13.44) |
|  | 1 day, > 2 PLT transfusions | NA* | NA* |
|  | 3 days, 1 to 2 PLT transfusions | 2.32 (0.60 to 9.01) | 1.98 (0.49 to 8.02) |
|  | 3 days, > 2 PLT transfusions | 8.12 (0.80 to 82.2) | 7.28 (0.69 to 77.06) |
|  | 5 days, 1 to 2 PLT transfusions | 2.16 (0.37 to 12.55) | 2.18 (0.37 to 12.68) |
|  | 5 days, > 2 PLT transfusions | 13.11 (1.91 to 90.03) | 11.18 (1.55 to 80.85) |
|  | 7 days, 1 to 2 PLT transfusions | 4.04 (0.73 to 22.27) | 4.06 (0.73 to 22.40) |
|  | 7 days, > 2 PLT transfusions | 8.91 (1.53 to 51.73) | 8.53 (1.34 to 54.42) |
|  |  |  |  |
|  |  |  |  |
| **Measurement of platelet transfusion** | **Index period** | **Crude** | **Adjusted for infection treatment**  **in index period (Yes/No)** |
| **Platelet transfusion, continuous** | |  |  |
|  | 1 day | 2.46 (1.02 to 5.91) | 2.66 (1.08 to 6.52) |
|  | 3 days | 1.92 (1.10 to 3.36) | 1.96 (1.10 to 3.51) |
|  | 5 days | 1.60 (1.08 to 2.37) | 1.55 (1.03 to 2.34) |
|  | 7 days | 1.48 (1.06 to 2.07) | 1.43 (1.02 to 2.02) |
| **Platelet transfusion, categorical (ref=0)** | |  |  |
|  | 1 day, 1 to 2 PLT transfusions | 3.86 (1.08 to 13.79) | 4.23 (1.17 to 15.37) |
|  | 1 day, > 2 PLT transfusions | NA* | NA* |
|  | 3 days, 1 to 2 PLT transfusions | 2.32 (0.60 to 9.01) | 2.35 (0.60 to 9.22) |
|  | 3 days, > 2 PLT transfusions | 8.12 (0.80 to 82.2) | 8.39 (0.76 to 93.04) |
|  | 5 days, 1 to 2 PLT transfusions | 2.16 (0.37 to 12.55) | 1.91 (0.32 to 11.26) |
|  | 5 days, > 2 PLT transfusions | 13.11 (1.91 to 90.03) | 11.57 (1.62 to 82.49) |
|  | 7 days, 1 to 2 PLT transfusions | 4.04 (0.73 to 22.27) | 3.52 (0.61 to 20.20) |
|  | 7 days, > 2 PLT transfusions | 8.91 (1.53 to 51.73) | 7.21 (1.16 to 44.89) |
| **Measurement of platelet transfusion** | **Index period** | **Crude** | **Adjusted for maximum leukocyte counts (ref = <10; median/IQR)** |
| **Platelet transfusion, continuous** | |  |  |
|  | 1 day | 2.46 (1.02 to 5.91) | 1.53 (0.56 to 4.19) of NA? |
|  | 3 days | 1.92 (1.10 to 3.36) | 1.87 (1.07 to 3.28) |
|  | 5 days | 1.60 (1.08 to 2.37) | 1.57 (1.05 to 2.36) |
|  | 7 days | 1.48 (1.06 to 2.07) | 1.50 (1.06 to 2.11) |
| **Platelet transfusion, categorical (ref=0)** | |  |  |
|  | 1 day, 1 to 2 PLT transfusions | 3.86 (1.08 to 13.79) | 1.22 (0.25 to 5.96) |
|  | 1 day, > 2 PLT transfusions | NA* | NA* |
|  | 3 days, 1 to 2 PLT transfusions | 2.32 (0.60 to 9.01) | 2.17 (0.56 to 8.43) |
|  | 3 days, > 2 PLT transfusions | 8.12 (0.80 to 82.2) | 7.87 (0.78 to 79.52) |
|  | 5 days, 1 to 2 PLT transfusions | 2.16 (0.37 to 12.55) | 2.07 (0.33 to 12.92) |
|  | 5 days, > 2 PLT transfusions | 13.11 (1.91 to 90.03) | 13.71 (1.77 to 105.81) |
|  | 7 days, 1 to 2 PLT transfusions | 4.04 (0.73 to 22.27) | 4.23 (0.72 to 24.94) |
|  | 7 days, > 2 PLT transfusions | 8.91 (1.53 to 51.73) | 9.47 (1.55 to 57.62) |
| **Measurement of platelet transfusion** | **Index period** | **Crude** | **Adjusted for non to intracranial bleeding events in index period (Yes/No)** |
| **Platelet transfusion, continuous** | |  |  |
|  | 1 day | 2.46 (1.02 to 5.91) | 2.42 (0.92 to 6.39) |
|  | 3 days | 1.92 (1.10 to 3.36) | 1.76 (0.97 to 3.19) |
|  | 5 days | 1.60 (1.08 to 2.37) | 1.45 (0.98 to 2.14) |
|  | 7 days | 1.48 (1.06 to 2.07) | 1.34 (0.98 to 1.83) |
| **Platelet transfusion, categorical (ref=0)** | |  |  |
|  | 1 day, 1 to 2 PLT transfusions | 3.86 (1.08 to 13.79) | 3.24 (0.84 to 12.43) |
|  | 1 day, > 2 PLT transfusions | NA* | NA* |
|  | 3 days, 1 to 2 PLT transfusions | 2.32 (0.60 to 9.01) | 1.66 (0.41 to 6.70) |
|  | 3 days, > 2 PLT transfusions | 8.12 (0.80 to 82.2) | 4.37 (0.38 to 49.80) |
|  | 5 days, 1 to 2 PLT transfusions | 2.16 (0.37 to 12.55) | 1.31 (0.21 to 8.33) |
|  | 5 days, > 2 PLT transfusions | 13.11 (1.91 to 90.03) | 8.70 (1.20 to 62.95) |
|  | 7 days, 1 to 2 PLT transfusions | 4.04 (0.73 to 22.27) | 2.98 (0.52 to 17.20) |
|  | 7 days, > 2 PLT transfusions | 8.91 (1.53 to 51.73) | 5.54 (0.92 to 33.27) |
| **Measurement of platelet transfusion** | **Index period** | **Crude** | **Adjusted for trauma (Yes/No; e.g. fall, interventions like LP or line placement)** |
| **Platelet transfusion, continuous** | |  |  |
|  | 1 day | 2.46 (1.02 to 5.91) | 3.25 (1.18 to 8.93) |
|  | 3 days | 1.92 (1.10 to 3.36) | 2.89 (1.32 to 6.36) |
|  | 5 days | 1.60 (1.08 to 2.37) | 1.90 (1.19 to 3.03) |
|  | 7 days | 1.48 (1.06 to 2.07) | 1.47 (1.05 to 2.06) |
| **Platelet transfusion, categorical (ref=0)** | |  |  |
|  | 1 day, 1 to 2 PLT transfusions | 3.86 (1.08 to 13.79) | 4.94 (1.24 to 19.61) |
|  | 1 day, > 2 PLT transfusions | NA* | NA* |
|  | 3 days, 1 to 2 PLT transfusions | 2.32 (0.60 to 9.01) | NA* |
|  | 3 days, > 2 PLT transfusions | 8.12 (0.80 to 82.2) | NA* |
|  | 5 days, 1 to 2 PLT transfusions | 2.16 (0.37 to 12.55) | 3.22 (0.46 to 22.47) |
|  | 5 days, > 2 PLT transfusions | 13.11 (1.91 to 90.03) | 38.83 (3.29 to 458.98) |
|  | 7 days, 1 to 2 PLT transfusions | 4.04 (0.73 to 22.27) | 3.47 (0.63 to 19.20) |
|  | 7 days, > 2 PLT transfusions | 8.91 (1.53 to 51.73) | 7.61 (1.34 to 43.57) |
|  |  |  |  |
|  |  |  |  |
| **Measurement of platelet transfusion** | **Index period** | **Crude** | **Adjusted for lumbar puncture**  **(*Yes or no)*** |
| **Platelet transfusion, continuous** | |  |  |
|  | 1 day | 2.46 (1.02 to 5.91) | NA* |
|  | 3 days | 1.92 (1.10 to 3.36) | NA* |
|  | 5 days | 1.60 (1.08 to 2.37) | 1.65 (1.08 to 2.51) |
|  | 7 days | 1.48 (1.06 to 2.07) | 1.50 (1.06 to 2.11) |
| **Platelet transfusion, categorical (ref=0)** | |  |  |
|  | 1 day, 1 to 2 PLT transfusions | 3.86 (1.08 to 13.79) | NA* |
|  | 1 day, > 2 PLT transfusions | NA* | NA* |
|  | 3 days, 1 to 2 PLT transfusions | 2.32 (0.60 to 9.01) | NA* |
|  | 3 days, > 2 PLT transfusions | 8.12 (0.80 to 82.2) | NA* |
|  | 5 days, 1 to 2 PLT transfusions | 2.16 (0.37 to 12.55) | 2.43 (0.39 to 15.07) |
|  | 5 days, > 2 PLT transfusions | 13.11 (1.91 to 90.03) | 16.33 (2.13 to 124.95) |
|  | 7 days, 1 to 2 PLT transfusions | 4.04 (0.73 to 22.27) | 3.85 (0.69 to 21.49) |
|  | 7 days, > 2 PLT transfusions | 8.91 (1.53 to 51.73) | 8.90 (1.61 to 49.25) |
| **Measurement of platelet transfusion** | **Index period** | **Crude** | **Adjusted for fall**  **(Yes/No)** |
| **Platelet transfusion, continuous** | |  |  |
|  | 1 day | 2.46 (1.02 to 5.91) | 3.48 (1.22 to 9.92) |
|  | 3 days | 1.92 (1.10 to 3.36) | 2.95 (1.32 to 6.60) |
|  | 5 days | 1.60 (1.08 to 2.37) | 2.15 (1.21 to 3.83) |
|  | 7 days | 1.48 (1.06 to 2.07) | 1.65 (1.10 to 2.46) |
| **Platelet transfusion, categorical (ref=0)** | |  |  |
|  | 1 day, 1 to 2 PLT transfusions | 3.86 (1.08 to 13.79) | NA* |
|  | 1 day, > 2 PLT transfusions | NA* | NA* |
|  | 3 days, 1 to 2 PLT transfusions | 2.32 (0.60 to 9.01) | 2.94 (0.68 to 12.82) |
|  | 3 days, > 2 PLT transfusions | 8.12 (0.80 to 82.2) | NA* |
|  | 5 days, 1 to 2 PLT transfusions | 2.16 (0.37 to 12.55) | 2.36 (0.38 to 14.71) |
|  | 5 days, > 2 PLT transfusions | 13.11 (1.91 to 90.03) | 28.62 (2.60 to 315.03) |
|  | 7 days, 1 to 2 PLT transfusions | 4.04 (0.73 to 22.27) | 3.95 (0.72 to 21.81) |
|  | 7 days, > 2 PLT transfusions | 8.91 (1.53 to 51.73) | 9.15 (1.57 to 53.44) |
| **Measurement of platelet transfusion** | **Index period** | **Crude** | **Adjusted for vomiting in index period (Yes/No)** |
| **Platelet transfusion, continuous** | |  |  |
|  | 1 day | 2.46 (1.02 to 5.91) | 2.41 (1.00 to 5.78) |
|  | 3 days | 1.92 (1.10 to 3.36) | 1.93 (1.09 to 3.39) |
|  | 5 days | 1.60 (1.08 to 2.37) | 1.55 (1.02 to 2.35) |
|  | 7 days | 1.48 (1.06 to 2.07) | 1.46 (1.03 to 2.07) |
| **Platelet transfusion, categorical (ref=0)** | |  |  |
|  | 1 day, 1 to 2 PLT transfusions | 3.86 (1.08 to 13.79) | 3.73 (1.03 to 13.50) |
|  | 1 day, > 2 PLT transfusions | NA* | NA* |
|  | 3 days, 1 to 2 PLT transfusions | 2.32 (0.60 to 9.01) | 2.29 (0.59 to 8.97) |
|  | 3 days, > 2 PLT transfusions | 8.12 (0.80 to 82.2) | 7.65 (0.74 to 79.58) |
|  | 5 days, 1 to 2 PLT transfusions | 2.16 (0.37 to 12.55) | 1.98 (0.32 to 12.40) |
|  | 5 days, > 2 PLT transfusions | 13.11 (1.91 to 90.03) | 11.15 (1.36 to 91.44) |
|  | 7 days, 1 to 2 PLT transfusions | 4.04 (0.73 to 22.27) | 3.81 (0.68 to 21.21) |
|  | 7 days, > 2 PLT transfusions | 8.91 (1.53 to 51.73) | 7.86 (1.31 to 47.25) |
| **Measurement of platelet transfusion** | **Index period** | **Crude** | **Adjusted for number of RBC**  **transfusions (median/IQR)** |
| **Platelet transfusion, continuous** | |  |  |
|  | 1 day | 2.46 (1.02 to 5.91) | 2.46 (1.02 to 5.91) |
|  | 3 days | 1.92 (1.10 to 3.36) | 1.93 (1.07 to 3.47) |
|  | 5 days | 1.60 (1.08 to 2.37) | 1.92 (1.08 to 3.39) |
|  | 7 days | 1.48 (1.06 to 2.07) | 1.55 (1.05 to 2.29) |
| **Platelet transfusion, categorical (ref=0)** | |  |  |
|  | 1 day, 1 to 2 PLT transfusions | 3.86 (1.08 to 13.79) | 3.87 (1.08 to 13.81) |
|  | 1 day, > 2 PLT transfusions | NA* | NA* |
|  | 3 days, 1 to 2 PLT transfusions | 2.32 (0.60 to 9.01) | 2.35 (0.55 to 10.12) |
|  | 3 days, > 2 PLT transfusions | 8.12 (0.80 to 82.2) | 8.28 (0.72 to 95.73) |
|  | 5 days, 1 to 2 PLT transfusions | 2.16 (0.37 to 12.55) | 2.72 (0.41 to 17.96) |
|  | 5 days, > 2 PLT transfusions | 13.11 (1.91 to 90.03) | 21.74 (1.69 to 279.28) |
|  | 7 days, 1 to 2 PLT transfusions | 4.04 (0.73 to 22.27) | 3.87 (0.69 to 21.68) |
|  | 7 days, > 2 PLT transfusions | 8.91 (1.53 to 51.73) | 7.99 (1.29 to 49.48) |
|  |  |  |  |
|  |  |  |  |
| **Measurement of platelet transfusion** | **Index period** | **Crude** | **Adjusted for**  **RBC transfusions categorical** |
| **Platelet transfusion, continuous** | |  |  |
|  | 1 day | 2.46 (1.02 to 5.91) | 2.45 (1.01 to 5.96) |
|  | 3 days | 1.92 (1.10 to 3.36) | 1.78 (0.95 to 3.33) |
|  | 5 days | 1.60 (1.08 to 2.37) | 1.96 (1.15 to 3.34) |
|  | 7 days | 1.48 (1.06 to 2.07) | 1.56 (1.07 to 2.28) |
| **Platelet transfusion, categorical (ref=0)** | |  |  |
|  | 1 day, 1 to 2 PLT transfusions | 3.86 (1.08 to 13.79) | 3.82 (1.06 to 13.75) |
|  | 1 day, > 2 PLT transfusions | NA* | NA* |
|  | 3 days, 1 to 2 PLT transfusions | 2.32 (0.60 to 9.01) | 2.23 (1.54 to 9.13) |
|  | 3 days, > 2 PLT transfusions | 8.12 (0.80 to 82.2) | 4.86 (0.41 to 57.41) |
|  | 5 days, 1 to 2 PLT transfusions | 2.16 (0.37 to 12.55) | 3.95 (0.44 to 35.75) |
|  | 5 days, > 2 PLT transfusions | 13.11 (1.91 to 90.03) | 65.83 (2.67 to 1623.06) |
|  | 7 days, 1 to 2 PLT transfusions | 4.04 (0.73 to 22.27) | 4.67 (0.75 to 29.15) |
|  | 7 days, > 2 PLT transfusions | 8.91 (1.53 to 51.73) | 11.18 (1.60 to 78.31) |

*NA: conditional logistic regression model did not run due to no disconcordant groups or too little events
